# The Elbe Estuary Microbiome Shifts With Salinity and Discharge and Depends on Fresh Organic Matter and Nutrient Availability

**DOI:** 10.1111/1758-2229.70349

**Published:** 2026-04-28

**Authors:** Vanessa Russnak, Raphael Koll, Sabine Keuter, Tina Sanders, Kirstin Dähnke

**Affiliations:** ^1^ Helmholtz‐Centre Hereon, Institute of Carbon Cycles Geesthacht Germany; ^2^ Institute of Marine Ecosystem and Fishery Science, University of Hamburg Hamburg Germany

**Keywords:** discharge, estuary, microbial community, seasonal variation, spatial variations

## Abstract

The Elbe Estuary (Germany) stretches 142 km from the weir in Geesthacht to the North Sea. It is classified as mesotidal, partially mixed and heavily impacted by anthropogenic activities and modifications. Despite well‐documented changes in ecosystem status, little is known about the microbial community in its surface water. In this study, we used 16S rDNA sequencing to characterise bacterial communities in surface water of the Elbe Estuary. Samples were collected across three seasons (winter, spring, and summer) in 2021 and 2022, to assess the relationship between environmental factors and bacterial community structure. Our analyses revealed that bacterial community diversity and composition varied seasonally and along the estuary stretch and were closely linked to physicochemical properties. Alpha diversity was highest in winter and in oligohaline samples. Distance‐based redundancy analysis showed that salinity, discharge, temperature, inorganic nitrogen (NO_2_), and silicate are key factors in shaping the bacterial community compositions. Although spatial differences were observed, seasonal variation was the main determinant of bacterial diversity and community structure. Overall, our results show that anthropogenic pressures and seasonal changes are reflected in a dynamic microbial community with metabolic functions strongly shaped by human activity.

## Graphical abstract

Microbial diversity within the Elbe Estuary was found to be more strongly governed by seasonal variability, salinity, and discharge than by spatial heterogeneity. Oligohaline regions sustain high diversity, while nutrient and redox conditions drive functional shifts. The estuarine community proves dynamic and sensitive to future environmental change.

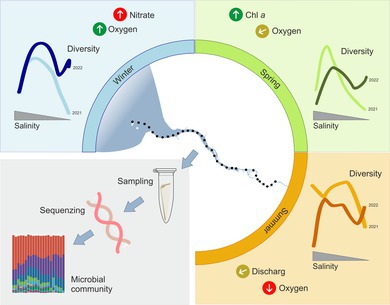

## 1 Introduction

Estuarine ecosystems are highly productive and dynamic transition zones, between limnic and marine systems, where river freshwater meets and intermixes with saline seawater (Bianchi 2006). Transition zones have unique physical and biogeochemical characteristics. The combined effect of nutrient dynamics, hydrological conditions and biological interactions is crucial in determining ecological processes and influences biogeochemical cycles and the habitats of diverse organisms (Hoitink and Jay 2016). The mixing of riverine freshwater and saline seawater creates spatial gradients and temporal variability in salinity, nutrients, and turbidity (Cloern et al. 2017). These gradients are not static and shift, leading to dynamic habitat boundaries across upper, middle, and lower estuarine zones (Day et al. 2012; Elliott and McLusky 2002). Estuaries are buffer zones that filter nutrients, sequester organic matter, and support diverse aquatic life (Jeffries et al. 2016; Kennish 2002). Riverine input to coastal zones also influences sediment and nutrient fluxes, productivity, and climate‐relevant processes such as greenhouse gas uptake and release (Moftakhari et al. 2013; O'Connor et al. 2022; Raymond et al. 2013). Rivers function as “active pipes” by processing materials when discharge is low and as “passive pipes” by quickly transporting materials downstream during periods of high discharge and turbidity, affecting nutrient distribution and ecosystem dynamics (Raymond et al. 2016). These hydrological dynamics shape microbial communities, which respond rapidly to changes in environmental variables (Crump et al. 2004; Wang et al. 2021).

Microbial communities are key regulators of biogeochemical processes like nutrient cycling and organic matter degradation within estuaries (Hall et al. 2018). Their composition, diversity, and activity are influenced by a variety of factors including salinity, nutrient availability, hydrodynamics, and anthropogenic pressures such as land use change and climate variability (Fortunato and Crump 2011; Kirchman et al. 2005). Riverine microbial communities are often dominated by a relatively limited number of phyla, including Actinobacteria, Proteobacteria, Bacteroidota, and Cyanobacteria (Amadei Martínez et al. 2025; Kang et al. 2017; Newton et al. 2007).

This study focuses on the Elbe Estuary, a mesotidal estuary in Northern Germany. The estuary has undergone major transformation due to variable anthropogenic pressure. Between 1985 and 2018, the estuarine status changed from polluted towards a recovery status, reflecting improved water quality and shifts in inorganic carbon, oxygen, and pH (Rewrie et al. 2023). Seasonal variability, rather than estuarine gradients or particle dynamics, has been identified as the primary driver of carbon distribution (Tobias‐Hünefeldt et al. 2024). Phytoplankton is year‐round dominated by mixotrophic flagellates and picophytoplankton (*Minidiscus* and *Mychonastes*), adapted to the estuary's turbid, low‐light, and variable‐salinity conditions (Martens et al. 2024). Zooplankton shows high trophic plasticity and adjusts feeding to organic matter availability (Biederbick et al. 2025). Organic‐rich aggregates provide microbial habitats and enhance trophic interactions, particularly in spring and summer (Zimmermann‐Timm et al. 1998). In addition, nitrification hotspots have been identified in the freshwater area downstream from the Hamburg port (Sanders et al. 2018).

Overall, a range of previous studies investigated nutrient and carbon fluxes in the Elbe Estuary and community composition in higher trophic levels, but comprehensive analyses of microbial community structure across spatial and seasonal scales remain limited. To bridge this gap, we conducted six sampling campaigns in 2021 and 2022, capturing key seasonal variations in microbial composition across the full salinity gradient. Our study aims to improve understanding of microbial ecology in the Elbe Estuary. Specifically, we (1) establish a baseline dataset of microbial community composition across surface waters over multiple seasons, (2) identify dominant and unique microbial taxa and address them, and (3) determine key environmental drivers that shape microbial community structure and dynamics. On this basis, we strive to understand how microbial communities respond to environmental variability in mesotidal estuaries and provide a foundation for long‐term monitoring and ecosystem modelling under future climatic and anthropogenic changes.

## 2 Material and Method

### 2.1 Study Area and Sampling

**FIGURE 1:**
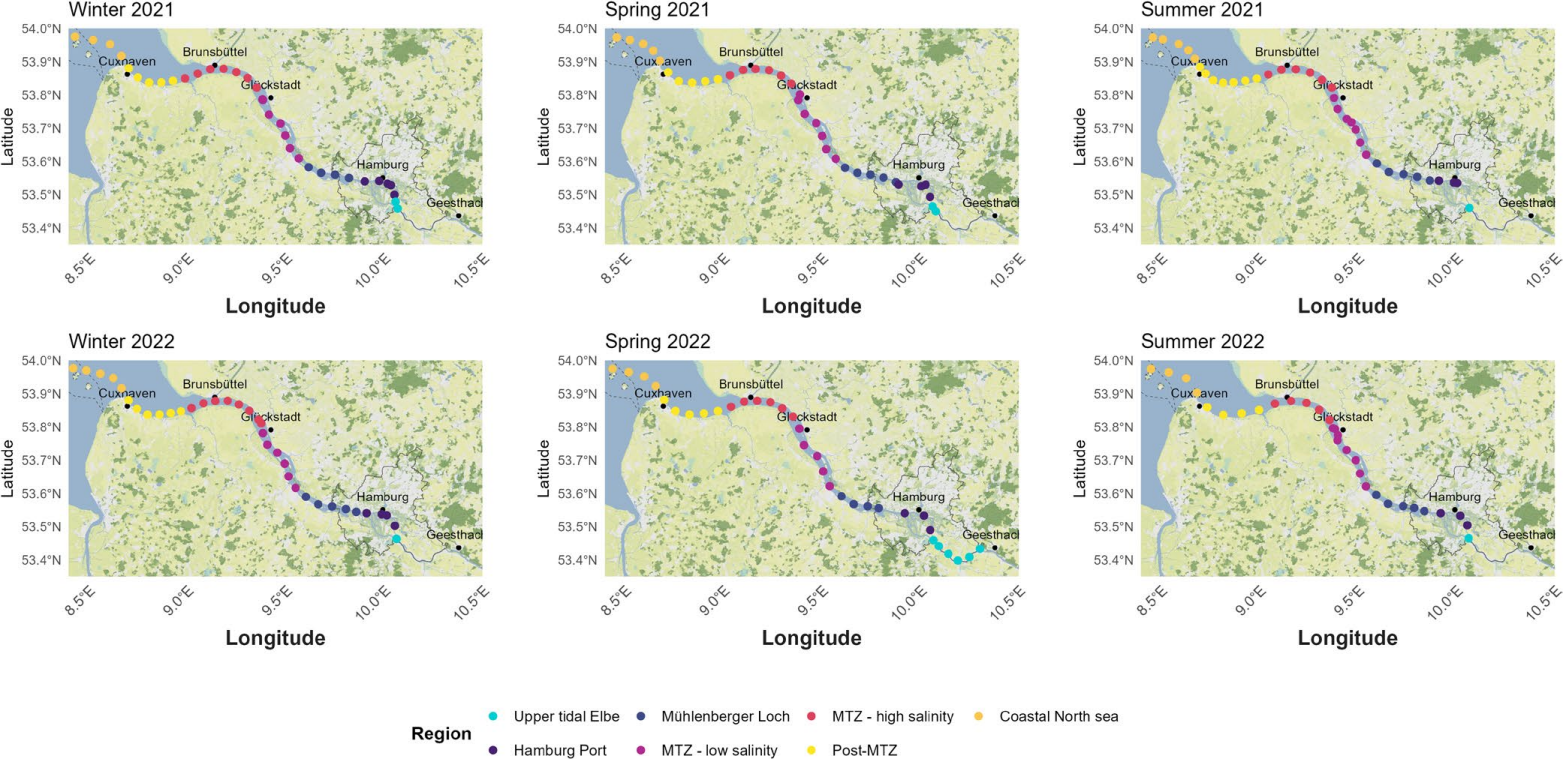
Map of the Elbe Estuary, dots indicate the different sampling points during our six different research cruises. Colour indicating the seven zones along the estuary adjusted after (Geerts et al. 2012). With the following region: Upper tidal Elbe, Hamburg Port, Mühlenberger Loch, MTZ low salinity, MTZ high salinity, Post‐MTZ, Coastal North Sea.

The present study was conducted in the Elbe Estuary (Figure 1), which connects the Elbe River to the North Sea. The Elbe River has a watershed of 148,268 km^2^, a population of ~24.4 million, and a mean annual river discharge of 653.2 m^3^/s (measured at Neu Darchau, stream km 536; Schulz, van Beusekom, et al. 2023). The estuary extends over 142 km from the weir at Geesthacht (Elbe‐km 585) to Cuxhaven (Elbe‐km 727), at which point it enters the North Sea (Boehlich and Strotmann 2019). The main channel is heavily dredged to enable access to the Port of Hamburg (Elbe‐km 613–628), the third largest port with overseas traffic in Europe. The tidal range of the estuary varies from 2 m at the weir to 3.5 m in the port area. The water column is partially well‐mixed (Pein et al. 2021), and the residence time varies between two to four weeks, depending on river discharge (Amann et al. 2012). The maximum turbidity zone (MTZ), an area of intense particle accumulation and mixing (Boehlich and Strotmann 2008), is located around Glückstadt (Elbe‐km 674) and extends ~30 km up and downstream the estuary (Geerts et al. 2012; Papenmeier et al. 2014).

For this study, the Elbe Estuary was divided into seven distinct regions, based on (Geerts et al. 2012): upper tidal Elbe, Hamburg Port, Mühlenberger Loch, MTZ low salinity, MTZ high salinity, Post‐MTZ, and coastal North Sea (Figure 1 and Table 1). Samples were categorised into five water types based on salinity: freshwater (salinity < 0.5), oligohaline (> 0.5 to < 5), mesohaline (> 5 to < 18), polyhaline (> 18 to < 30) and marine water (salinity > 30). Due to the limited sample size of the marine end‐member (*n* = 2), these data were pooled with polyhaline samples to create a high‐salinity composite group. This allows for a broader comparison against mesohaline and oligohaline reaches, but we explicitly acknowledge that this grouping limits our ability to disentangle true marine end‐member groups from subsets with estuarine influence.

**TABLE 1:** Zonation of Elbe estuary as adjusted after the TIDE project with corresponding region of estuary (Geerts et al. 2012).

Region	Abbreviation	km	Salinity
Upper tidal Elbe	A	585–612	**Limnic**, < 0.5
Hamburg Port	B	613–628	OMZ, **Limnic**, < 0.5
Mühlenberger Loch	C	629–650	OMZ, **Limnic**, < 0.5
MTZ low salinity	D	651–677	influence of brackish water at low discharge **Oligohaline**, 0.5–5
MTZ high salinity	E	678–704	brackish water zone, variable salinities **Mesohaline**, (> 5–18)
Post MTZ	F	705–727	Brackish water zone, variable salinities between **Mesohaline** (> 5–18) and occasionally polyhaline (> 30)
Coastal North Sea	G	728–760	**Polyhaline**, > 18–30

*Note:* Samples were categorised according to salinity (bold).

In 2021 and 2022, a total of six seasonal sampling campaigns were conducted with *R/V Ludwig Prandtl* (Table 2). Samples were taken on transects on two consecutive days against the outgoing tide in the main channel of the Elbe Estuary, from near Scharhörn island (~20 km off Cuxhaven) upstream to stream kilometre ~609, just beyond Hamburg Port. Dissolved oxygen, temperature, salinity, and pH were continuously measured through a FerryBox system (Petersen et al. 2011), with water being pumped from approximately 1.5 m depth. Additional water samples were taken every 20 min. For nutrient analysis, a defined water volume was filtered immediately through combusted, pre‐weighted GF/F filters (4 h, 450°C), and stored frozen (−20°C) until analysis. The filters were used for analysis of suspended particulate matter (SPM), particulate nitrogen (PN), particulate carbon (PC), and C/N ratio. For chlorophyll *a* (Chl *a*) analysis, a defined water volume was filtered through a GF/F filter, and the filter was immediately stored frozen. DNA samples were taken at least at every second station, adding up to approx. 20 samples per transect (Table 2). Water was filtered immediately through a 0.22 μm polycarbonate filter (Merck Millipore) until the filter was saturated (min. 250 mL). Filters were stored frozen (−20°C) until extraction in the laboratory. The spatial resolution of microbiome sampling thus was coarser than that of the continuously measured hydrochemical parameters.

**TABLE 2:** Campaign dates with the sampled Elbe Estuary sections shown via stream kilometres, averages and standard deviations for water temperature for each campaign, average discharge during each survey, measured at the Neu Darchau gauging station and the number of environmental measurements (Env) and samples used for DNA analysis.

Campaign dates	Season	Stream kilometres [km]	Water temperature [°C]	Average freshwater discharge [m^3^/s]	Sample Env/DNA
09–12 March 2021	Winter 2021	609–748	5.4 ± 0.5	862	34/21
04–05 May 2021	Spring 2021	610–751	10.5 ± 0.8	411	35/17
27–28 July 2021	Summer 2021	621–751	22.2 ± 0.7	721	35/25
01–02 March 2022	Winter 2022	610–752	5.6 ± 0.2	1282	36/21
22–25 May 2022	Spring 2022	588–752	17.8 ± 1.8	336	37/25
14–15 June 2022	Summer 2022	610–752	18.9 ± 1.3	241	31/20

### 2.2 Nutrient Measurements

Concentrations of nutrients (nitrate, nitrite, ammonium, silicate and phosphate) were measured with a continuous‐flow automatic analyser (AA3, SEAL Analytics) using standard colorimetric and fluorometric techniques (Hansen and Koroleff 2007). The detection limits were 0.05 μmol/L^−1^ for nitrate (NO_3_^−^), 0.05 μmol/L^−1^ for nitrite (NO_2_^−^), 0.07 μmol/L^−1^ for ammonium (NH_4_^+^), 0.03 μmol/L^−1^ for silicate (SiO_4_) and 0.13 μmol/L^−1^ for phosphate (PO_4_^3^). Particulate nitrogen and carbon content on the filters was measured by an Elemental Analyser (Eurovector EA 3000) calibrated against a certified acetanilide standard (IVA Analysentechnik, Germany). The standard deviation was 0.05% for carbon and 0.005% for nitrogen. Chlorophyll *a* (Chl *a*) was measured photometrically after extraction in 90% acetone following the protocol (Jeffrey and Humphrey 1975).

### 2.3 DNA Extraction and 16S Amplicon Sequencing

After thawing on ice, biomass was rinsed from the filter with distilled high‐purity 0.9% NaCl. The material was centrifuged and the supernatant discarded. The remaining pellet was used for DNA extraction using the DNeasy PowerSoil Pro Kit (QIAGEN GmbH, Hilden, Germany) according to the manufacturer's protocol. The sequencing and processing of the 16S rDNA variable regions V3‐V4 were performed at the Competence Centre for Genomic Analysis in Kiel, Germany, using the Illumina Nextera XT Index Kit and the primers 341F‐806R (dual‐barcoding approach, Kozich et al. 2013; primer sequences: 5′‐CCTACGGGAGGCAGCAG‐3′ (Muyzer and De Waal Uitierlinden 1993) and 5′‐GGACTACHVGGGTWTCTAAT‐3′ (Caporaso et al. 2011)). Cutadapt (v.4.4, Martin 2011) was used to trim the adapters of the demultiplexed paired‐end readings. The Hummel high‐performance cluster at the University of Hamburg was used for microbiota analysis. TrimGalore (v. 0.6.10) was used to filter the read files according to the adapter sequences, quality, and length after they had undergone quality control (Krueger 2015). The raw sequence data have been deposited in the ENA at EMBL‐EBI under the Project (PRJEB96293).

### 2.4 Amplicon Data Processing and Statistical Analysis

DADA2 (v.1.29.0) (Callahan et al. 2016) was used for amplicon sequence variant (ASV) prediction and taxonomy assignment using the SILVA SSU v138 taxonomic database (Quast et al. 2012). The ASV table and sample data were processed in the phyloseq package (v. 1.45.0) (McMurdie and Holmes 2011), removing ASVs that were taxonomically assigned to non‐bacterial groups including chloroplasts and archaea. To enhance interpretability and minimise the risk of spurious correlations, low‐abundance taxa in the dataset were filtered by a threshold of 0.001% of the total sum of counts, which strongly reduced the number of zeros in the dataset while minimally affecting overall counts (Figure S1).

All statistical analyses were conducted in R (version 4.4.1) using the visualisation packages *cowplot* (version 1.1.3) and ggplot2 (version 3.5.2). Alpha diversity metrics (Observed richness, Chao1, Shannon, and Pielou's evenness) were calculated from rarefied data using the *vegan* package (v. 2.6.4) (Oksanen et al. 2022). Samples with fewer than 5000 sequences (2 of 129) were removed prior to rarefaction, and remaining samples were rarefied to the minimum sequencing depth across the dataset (10,441 ± 2120, min. 5777 reads). To assess the robustness of the subsampling process, repeated rarefication (1000×) was performed and alpha diversity estimated were compared via Spearman correlations, confirming that the observed patterns were consistent across methods (Figure S2; see Methods S1, Supporting Information). Assumptions of normality (Shapiro–Wilk test) and homogeneity of variances (Fligner‐Killeen and Levene's tests) were assessed. As the assumptions of normality and homogeneity of variances were violated, non‐parametric statistical methods were applied. Differences in diversity metrics were assessed using the Kruskal‐Wallis rank‐sum test, followed by pairwise comparisons using Wilcoxon rank‐sum tests or Dunn's tests with Holm's correction for multiple testing. Spearman rank correlation analyses, implemented via the *Hmisc* package (version 5.2.3) (Harrell 2025), were applied to investigate correlations among environmental physicochemical parameters as well as to identify significant associations between the 40 most abundant genera and environmental factors. Principal coordinate analysis (PCoA) was then performed based on the pairwise averaged subsampled Bray–Curtis dissimilarity of surface samples (*n* = 127) to study how the samples cluster according to regions and season. The resulting distance matrix was further used to test for significant differences in community structure between the aforementioned factors using permutation analysis of variance (PERMANOVA) and ANOSIM.

Distance‐based redundancy analysis (dbRDA) was conducted using average Bray‐Curtis dissimilarities to analyse how communities responded to seasonal and spatial gradients. Prior to the analysis, all environmental variables were z‐transformed. Environmental and physiological variables were first selected through stepwise model building for constrained ordination using the *ordistep* function. To account for multicollinearity, variance inflation factors (VIFs) were calculated, and only variables with VIF < 7 among those selected by *ordistep* were retained as explanatory variables. The strength of association between these explanatory variables and the bacterial assemblage was then assessed by fitting environmental vectors onto the reduced dbRDA ordination using the *envfit* function from the *vegan* package. Due to high collinearity, the following parameters were excluded from the final model: turbidity, oxygen concentration, oxygen saturation, pH, suspended particulate matter (SPM), particulate carbon (PC), particulate nitrogen (PN), ammonium (NH_4_^+^), and nitrate (NO_3_^−^). Potential functions among bacteria were predicted by using FAPROTAX v1.2.7 (Louca et al. 2016). We used Corel Draw Graphics Suits 2022 for adjusting figures with additional text and creating the graphical abstract. An AI tool, ChatGPT (GPT‐4) was used to streamline R code and to check the finalised manuscript for common grammatical and typographical errors.

## 3 Results

### 3.1 Physical–Chemical Properties of Surface Water

**FIGURE 2:**
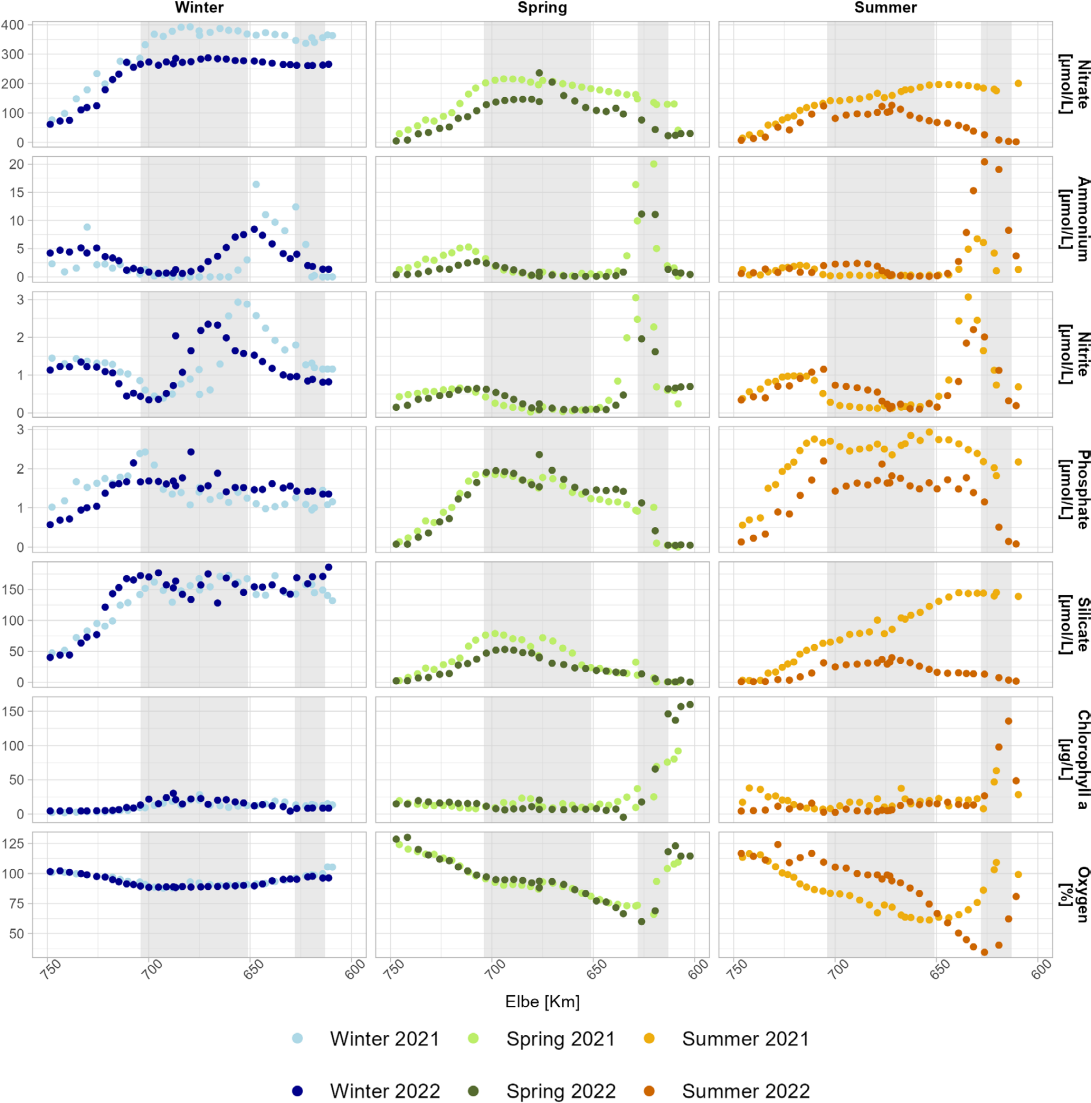
Longitudinal profiles of the main environmental parameters along the Elbe Estuary (Elbe‐km 750–600) during 2021 and 2022. Parameters include nitrate (μmol L^−1^), ammonium (μmol L^−1^), nitrite (μmol L^−1^), phosphate (μmol L^−1^), silicate (μmol L^−1^), chlorophyll *a* (μg L^−1^), and oxygen saturation (%). Data are grouped by season (winter, spring, and summer) and year, highlighting temporal and spatial variations. The grey highlighted areas are the Hamburg Port (613–628 km) and the MTZ (651–704 km).

Temperatures in the estuary ranged from 5.1°C in winter to 21.4°C in summer 2022 (Figure S3). Stream discharge varied between 241 and 1282 m^3^ s^−1^ during the sampling campaigns, with the highest discharge recorded in winter 2022 and the lowest in summer 2022 (Table 2). Salinity increased downstream, with steeper gradients in winter than in summer, reflecting the seasonal variation in discharge. Water turbidity reached its maximum (~300 mg L^−1^ SPM) between Elbe‐km 660 and 710 (Figure S3). The Elbe Estuary is characterised by high nutrient loads that change along the sampling stretch. The dominant dissolved inorganic nitrogen (DIN) compound during all six sampling campaigns was nitrate, with highest concentrations of 300–400 μmol L^−1^ observed in winter 2021/2022. Nitrate concentrations in the estuary and nitrate input from the riverine side decreased during spring and summer, which was especially evident in low nitrate concentrations of < 2 μmol L^−1^ in the upstream region in summer 2022 (Figure 2).

Ammonium and nitrite behaved similarly to each other across all seasons along the transect, with two distinct peaks along the sampling stretch. The first peak occurred in the limnic section, between Elbe‐km 620 and 630, where the streambed deepens from 10 to 18 m. Here, ammonium concentrations peaked in all seasons, ranging from 14–28 μmol L^−1^. A second peak was observed in the oligohaline section, in the maximum turbidity zone (MTZ). However, this downstream increase was less pronounced during spring and summer than in winter.

Oxygen concentrations in the upper tidal Elbe (Figure 2) were saturated (winter) or supersaturated (spring and summer) comparable to conditions in the North Sea. However, in the MTZ and especially the Hamburg Port region, dissolved oxygen in the water column decreased significantly in spring and summer, dropping from 80.9% to a minimum concentration of 34.5% over 16 km in summer 2022. In spring and summer, we measured high chlorophyll concentrations in the upper reaches of the Elbe (~140 μg L^−1^). Winter chlorophyll concentrations were low (~12 μg L^−1^) and slightly higher in the MTZ region, comparable to spring concentrations in the same area.

### 3.2 Diversity and Richness Analysis of Bacterial Communities

A total of 2,006,825 microbial sequence reads were obtained after quality filtering from 129 surface water samples. The sequences were clustered into 95,070 ASV (amplicon sequence variants). Prior to further analysis, two samples were removed due to low sequence reads output (< 5000).

**FIGURE 3:**
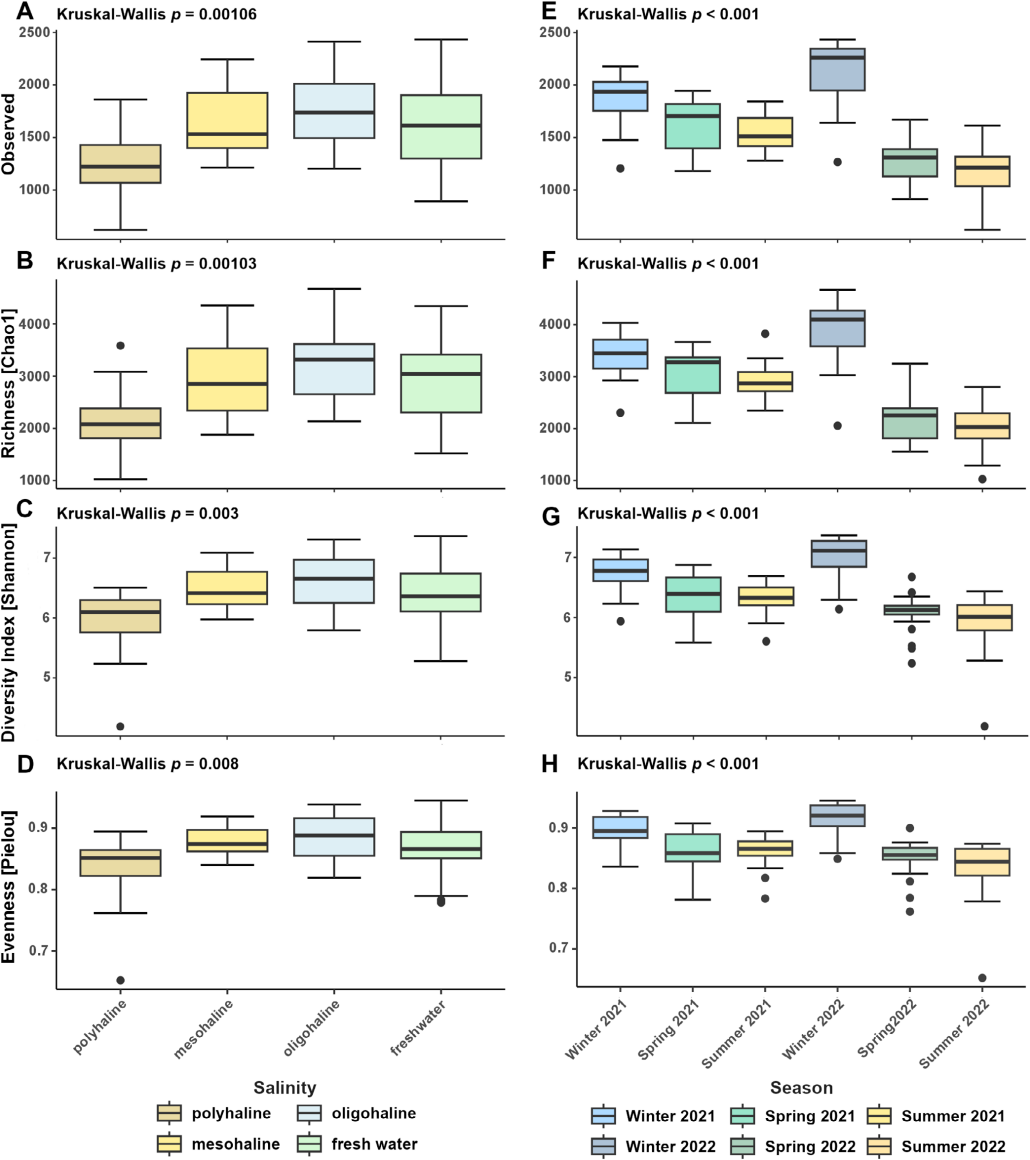
Estimates of Observed number, Richness Chao 1, Diversity Index Shannon and Pielou's Evenness for the bacterial community across salinity levels (A–D) and seasons (E–H).

Analysis of alpha diversity metrics revealed significant variation in microbial community structure across both salinity gradients and sampling campaigns (Figure 3). In general, polyhaline samples displayed lower numbers of observed taxa and Shannon diversity compared to other salinity categories (post hoc Dunn's test, Holm‐adjusted *p*‐values < 0.001, Table S1). Diversity increased from polyhaline to oligohaline conditions, with oligohaline sites exhibiting the highest richness and evenness. Notably, evenness was lowest in polyhaline and freshwater samples. Single campaigns compared with each other as well overall varied significantly from each other (Figure 3 E‐H). Richness and diversity were highest during winter 2021 and 2022, while spring and summer samples showed comparatively lower values. Evenness followed a similar pattern, displaying the lowest values during the summer and peaking in winter. Using a significance threshold of 0.001, no alpha diversity index differed significantly between the years 2021 and 2022 (Wilcoxon test, Holm‐adjusted *p*‐values > 0.001, Table S1), indicating temporal stability in microbial richness and evenness across seasons.

### 3.3 Bacterial Community Compositions in the Riverine‐Coastal System

**FIGURE 4:**
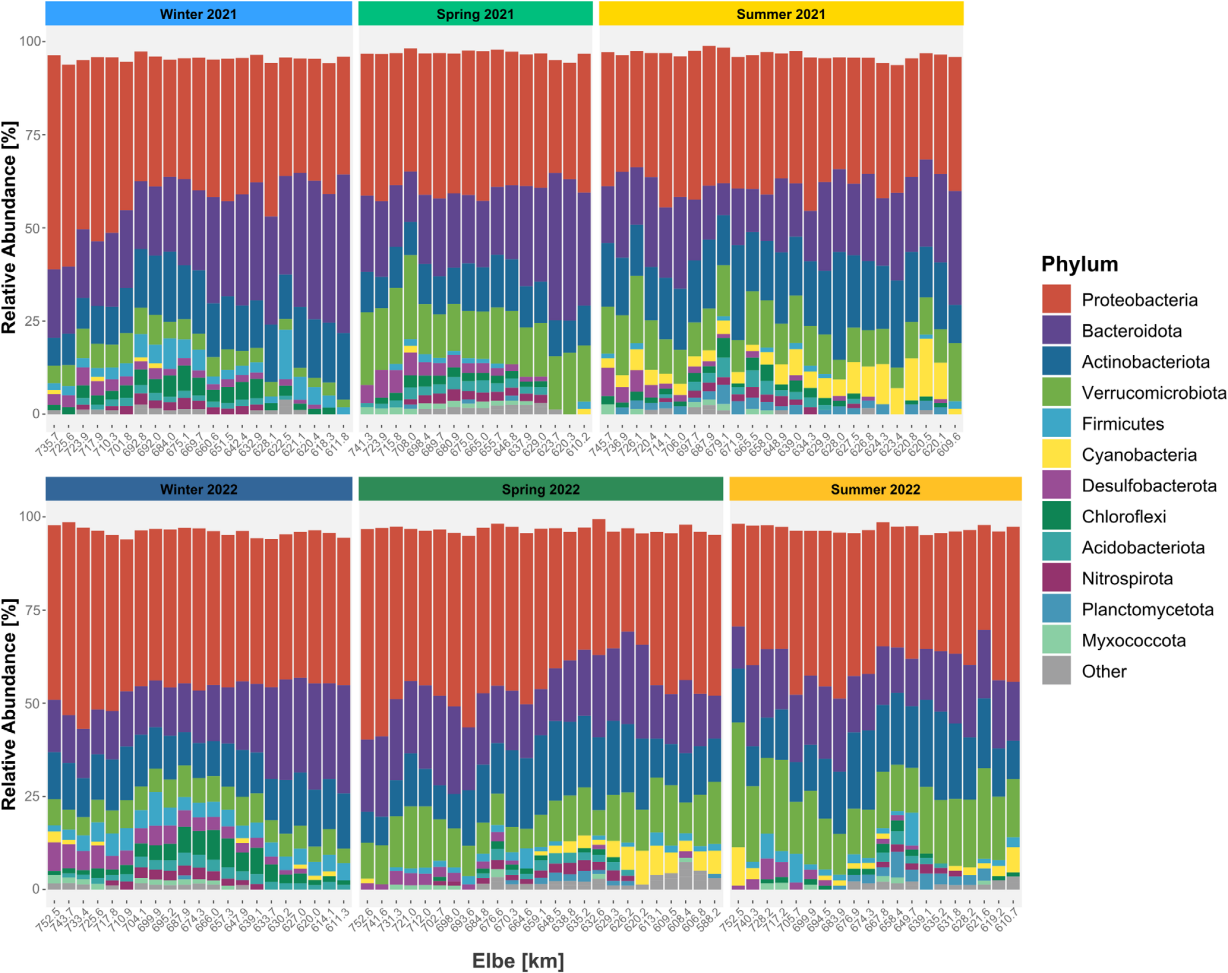
Barplot of relative abundance (%) of the dominant 12 phyla (> 1%) of the Elbe water in three seasons of 2021 and 2022 along the salinity gradient.

A total of 36 phyla were identified in the surface water samples. *Proteobacteria* (38.44%), *Actinobacteriota* (14.46%), *Bacteroidota* (19.89%), and *Verrucomicrobiota* (10.91%) were the most frequently detected phyla in all samples, accounting collectively for approximately 83.7% of the relative abundance (Figure 4). The phyla *Firmicutes* (up to 2%), *Cyanobacteria* (up to 2%), *Desulfobacterota* (up to 2%), and *Chloroflexi* (2%) constituted up to 9% of the relative abundance. Overall, the top 12 bacterial phyla accounted for 97.33% of the relative abundance of the bacterial sequences across all 127 water samples.

The relative abundance of the three main phyla Proteobacteria, Actinobacteriota, Bacteroidota is similar across all samples. Verrucomicrobiota is more strongly influenced by seasons, with a lower relative abundance in winter (up to 6%) compared to spring and summer (up to 17%). Firmicutes (up to 4%) and Chloroflexi (up to 3.5%) were more abundant in winter than in spring and summer, whereas Desulfobacterota and Cyanobacteria were more important in spring and summer, with a relative abundance of up to 5% each.

**FIGURE 5:**
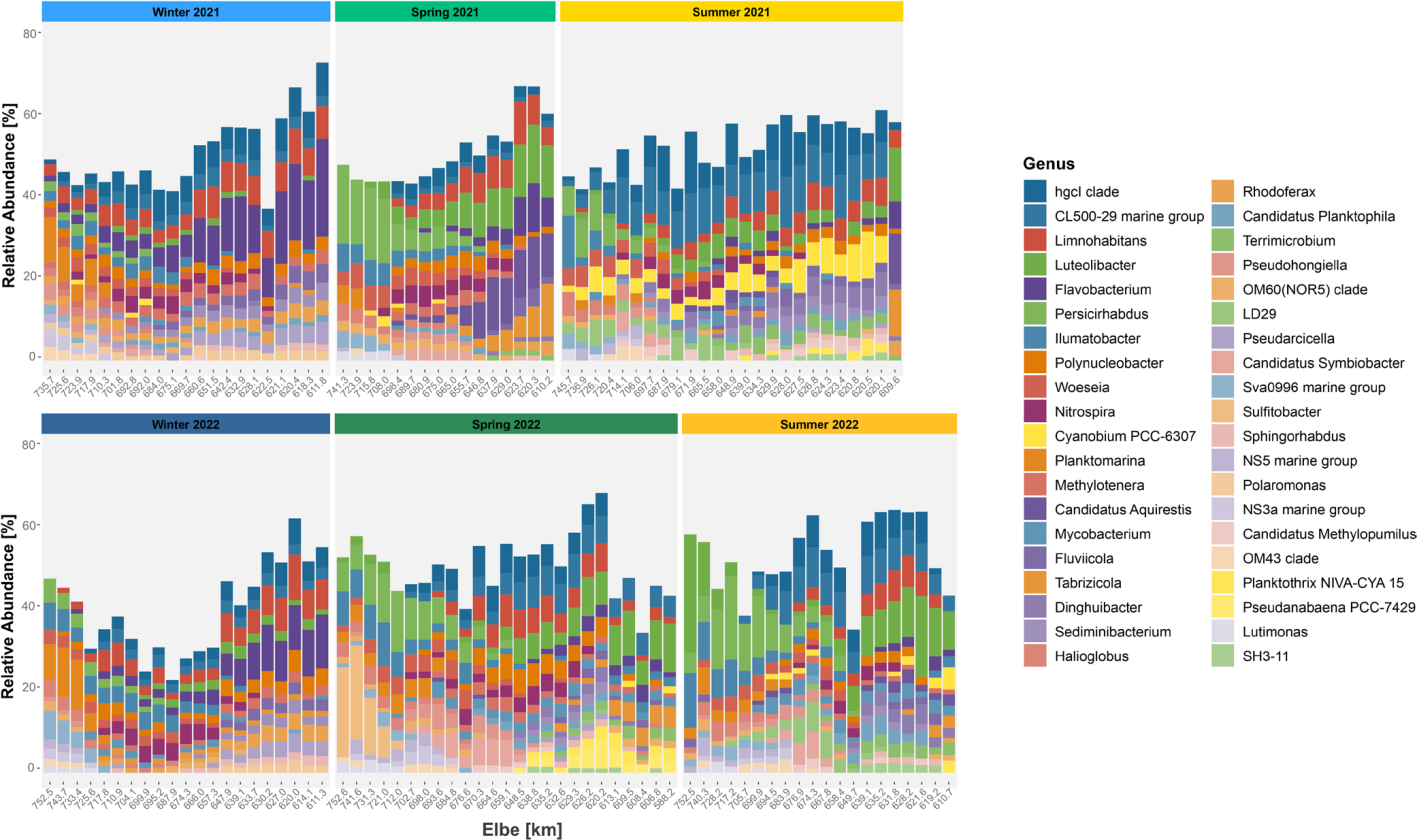
Barplot of relative abundance (%) of the dominant genera (> 1%) of the Elbe water in three seasons of 2021 and 2022 along the salinity gradient.

We identified 40 most abundant genera along the Elbe Estuary (Figure 5) in our sampling campaigns. These account for 38%–74% of all ASVs in 2021 and 23%–70% in 2022, whereas they represent a less variable percentage of all ASVs (~35%–65%) in spring and summer samplings. Among the top genera, 23 were consistently present in all seasons. These included *Limnohabitans* (Gammaproteobacteria), hgcI clade (Actinobacteriota), *Polynucleobacter* (Gammaproteobacteria), CL500‐29 marine group (Actinobacteriota), *Ilumatobacter* (Actinobacteriota), *Woeseia* (Gammaproteobacteria), and *Luteolibacter* (Verrucomicrobiota). The predominant genera were CL500‐29 marine group*, Limnohabitans*, the hgcI clade, alongside *Luteolibacter*, which each constituted approximately 4% of the total abundance of all samples. Furthermore, high abundances of region‐specific genera indicate spatial variability in community composition. A total of 17 genera occur only in certain sampling campaigns and are absent in others. As an example, *Sulfitobacter* and *Pseudanabaena PCC‐7429* were exclusively present in spring 2022.

Across both winter sampling campaigns, the bacterial community composition in the Elbe Estuary was comparable, with abundant taxa dominated by *Flavobacterium*, the hgcI clade, *Limnohabitans*, *Polynucleobacter*, and the CL500‐29 marine group. Alongside these dominant species, the community in the upper estuary (A–C) was primarily composed of the species *Pseudarcicella*, *Rhodoferax*, *Fluviicola*, and *Sediminibacterium*. Within the maximum turbidity zone (MTZ; D and E), *Nitrospira* reached a relative abundance of approximately 4%. In the North Sea region (F/G), dominant genera included *Planktomarina* (up to 11%), *Ilumatobacter* (up to 5%), *Persicirhabdus* (up to 4%), and *Woeseia* (up to 3%).

While both winter communities appeared relatively uniform across the estuarine gradient, a pronounced shift in the community composition appeared from winter to spring. Particularly in the upper estuary (A–C) and the marine‐influenced sites (F/G), the community structure changed. Despite this variability, the three genera *Luteolibacter*, CL500‐29 marine group, and *Limnohabitans* were consistently detected across all regions in spring. In the upper estuary (A–C), *Candidatus* Aquirestis (up to 12%), *Luteolibacter* (up to 13%), and *Tabrizicola* (13%) were among the most abundant taxa. In spring 2022, the cyanobacterial taxa *Pseudanabaena PCC‐7429* and *Planktothrix* NIVA‐CYA 15 reached high abundances (up to 5%) in the upper estuary region. Within the maximum turbidity zone (MTZ), the most prominent taxa were *Pseudohongiella*, *Candidatus* Symbiobacter, *Nitrospira*, and the hgcI clade. Towards the North Sea region (F/G), we found high abundances of *Persicirhabdus* (up to 19%) and *Ilumatobacter* (up to 8%). Interestingly, *Sulfitobacter* was exceptionally abundant in this marine‐influenced zone, reaching up to 19.9% relative abundance in spring 2022.

The summer samples of 2021 and 2022 display a similar shift in community composition along the estuary. Among all taxa, only *Luteolibacter* was consistently present in all regions in summer. The upper estuary (A–C) was primarily characterised by *Tabrizicola*, the CL500‐29 marine group, and *Limnohabitans*. The MTZ showed high microbial diversity, marked by an increase of saltwater‐associated taxa (respectively *Persicirhabdus* and *Woeseia*) as well as members of the *LD29* and *Nitrospira*. High relative abundance of *Cyanobium* PCC‐6307 (up to 7%) was reached during a cyanobacterial bloom in summer 2021. In contrast, no substantial cyanobacterial bloom occurred in summer 2022, although *Cyanobium* PCC‐6307 and *Planktothrix* NIVA‐CYA 15 were abundant in the upper estuary (A/B). In the North Sea region (F/G), the community was dominated by *Persicirhabdus*, *Ilumatobacter*, and *Halioglobus*, similar to findings in spring.

### 3.4 Correlations of Microbial Community With Physicochemical Parameters

**FIGURE 6:**
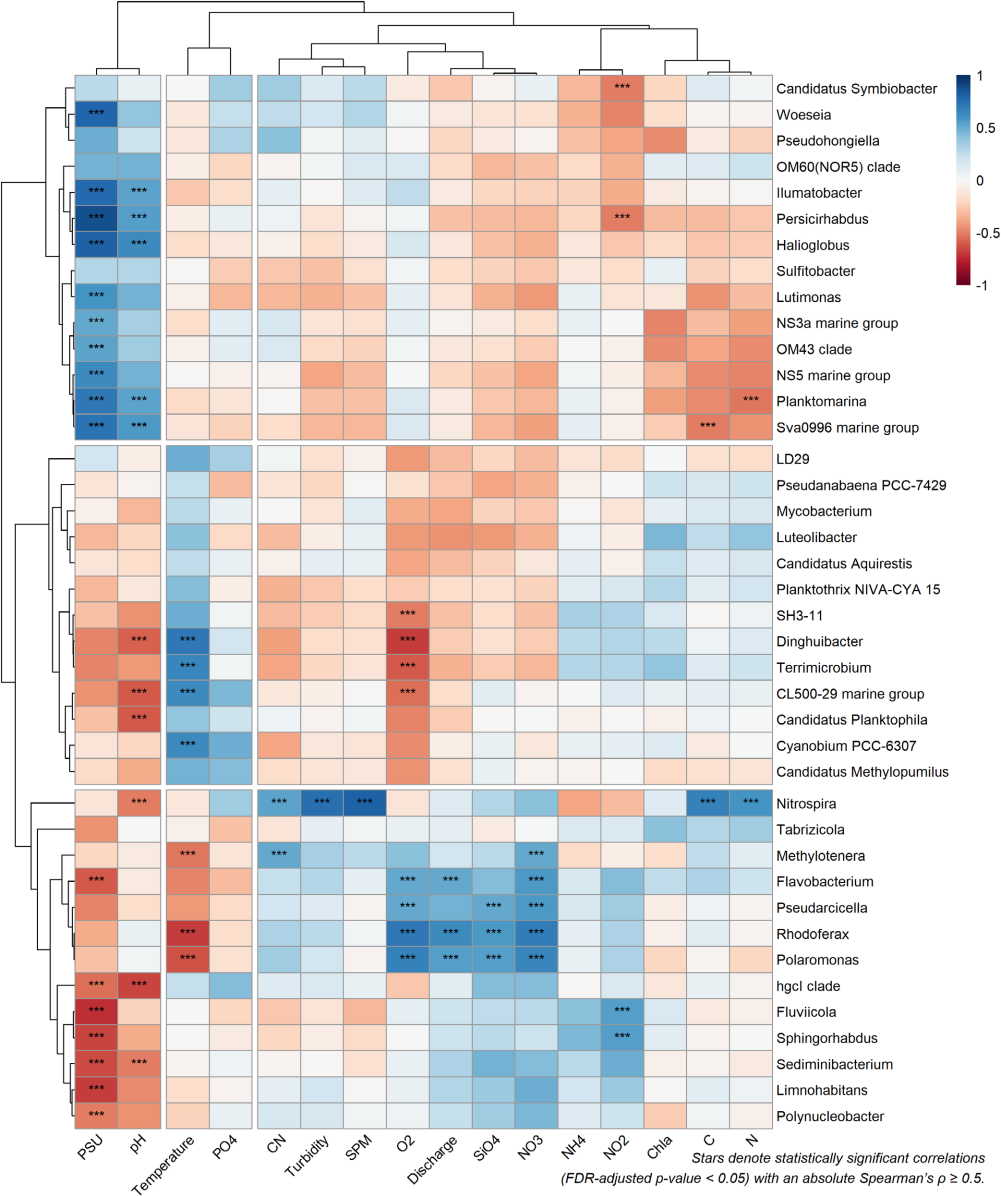
Spearman correlation heatmap based on the top 40 genera of the bacterial community and environmental variables. The X‐axis of the heatmap are the environmental factors and Y‐axis species at genus level. The colour scale on the right shows the colour partitioning of the different *R* values. Stars *** indicate FDR‐adjusted *p* ≤ 0.05 with an absolute Spearman's *p* > 0.5.

To identify correlations with physical and chemical water parameters across identified genera, we applied a Spearman correlation matrix (*ρ* ≥ |0.5|; FDR‐adjusted *p* < 0.05). Hierarchical cluster analysis of the resulting heat map revealed three distinct groups (see Figure 6).

One group of genera only showed a positive correlation with salinity and pH (Figure 6). In this group, the genera *Persicirhabdus* and *Candidatus* Symbiobacter were negatively correlated with nutrient concentrations (NO_2_^−^ and NH_4_^+^), while *Planktomarina* and the *Sva0996 marine group* were negatively associated with particulate carbon and nitrogen. The second group is predominantly characterised by species that show a positive correlation with temperature but negative correlations with pH and dissolved oxygen. Taxa in this cluster include CL500‐29 marine group, *Cyanobium* PCC‐6307, and *Dinghuibacter*. Genera in the third group were negatively correlated with salinity, pH, and temperature and positively correlated with dissolved oxygen, discharge, and nutrient concentrations (SiO_4_ and NO_3_^−^), such as the *hgcl clade*, *Sediminibacterium*, and *Limnohabitans*. Within this group, *Flavobacterium*, *Rhodoferax*, and *Methylotenera* are mainly positively correlated with oxygen, discharge, silicate, and nitrate. *Nitrospira* is distinct from all previously identified groups and shows positive correlations with dissolved organic carbon (CN), turbidity, and suspended particulate matter (SPM).

### 3.5 Driving Parameter of Beta Diversity

Beta diversity patterns of microbial communities were explored using principal coordinates analysis (PCoA), permutational multivariate analysis of variance (PERMANOVA), and analysis of similarities (ANOSIM). The PCoA revealed that the first two axes explained 33.7% of the variation in community composition (Figure S3). PERMANOVA further identified significant effects of salinity (Pseudo‐*F = 14.20, p = 0.001*), season (Pseudo‐*F = 7.28, p = 0.001*), and region (Pseudo‐*F = 8.83, p = 0.001*) on microbial community structure (Table S2).

PERMANOVA indicated a significant effect of year on community composition (Pseudo*‐F = 4.34, p = 0.001*). However, the betadisper test detected significant differences in group dispersions among years (*p* = *0.048*), indicating heterogeneity of multivariate variance. PERMANOVA can be sensitive to differences in dispersion; the observed year effect may partly reflect differences in within‐group variability rather than shifts in group centroids. Therefore, the influence of year should be interpreted cautiously and not necessarily as a strong directional change in community composition (Table S2).

Pairwise comparisons using FDR‐corrected *p*‐values revealed significant differences between all salinity levels (*p* = *0.001*). Most seasonal comparisons were also significant, particularly involving Spring 2022, which was distinct from Winter 2021, Spring 2021, Summer 2021, Winter 2022, and Summer 2022 (*p < 0.05*). Multiple regional comparisons were significant as well, with Region A differing from all other regions (*p ≤ 0.002*), and additional differences observed among sites C, G, F, E, and B. However, the significant differences in dispersion among groups suggest that heterogeneity of group variances may have influenced a few PERMANOVA results. ANOSIM confirmed clear group separation by both season (*R = 0.35, p = 0.001*) and salinity (*R = 0.50, p = 0.001*), reinforcing salinity as the primary environmental driver of microbial community composition in the Elbe Estuary.

**FIGURE 7:**
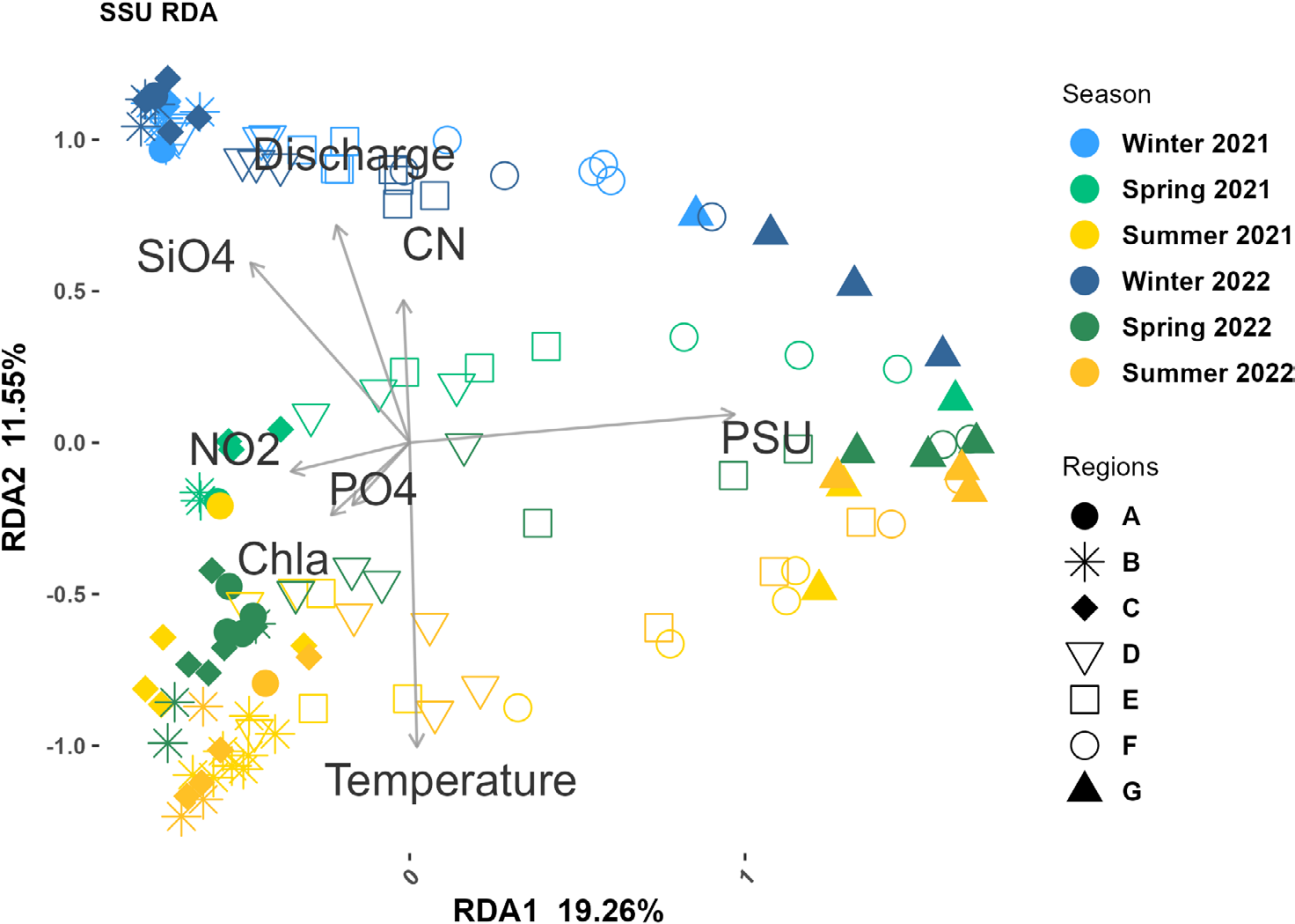
Distance‐based redundancy analysis (dbRDA) shows the relationship between physical–chemical and bacterial community.

Further, canonical ordination using distance‐based redundancy analysis (dbRDA) was applied (Figure 7), revealing that in addition to salinity and temperature, discharge and silicate were significant explanatory variables for microbial communities. The first two dbRDA axes explained 19.26% (RDA1) and 11.55% (RDA2) of the total variance, respectively. A seasonal effect was observed with a clear separation between the three different seasons. The temperature vector (*Pseudo‐F = 18.451, p < 0.001*) pointed towards the summer samples (A–D), while discharge (*Pseudo‐F = 19.747, p < 0.001*) and silicate (SiO_4_) (*Pseudo‐F = 7.757, p < 0.001*) vector pointed towards the upstream winter samples (A–D). Salinity emerged as the most significant environmental variable (*Pseudo‐F = 41.742, p < 0.001*) with a clear separation in freshwater and marine samples pointing towards all samples from the Region F‐G. Additionally, the vector of Chl *a*, NO_2_^−^ and PO_4_^3−^ oriented towards spring samples from Regions A‐C (Table S3). Notably, spring samples from 2021 and 2022 were also separated along the dbRDA axes, indicating interannual variation within the same season (Table S3).

### 3.6 Functional Prediction of Bacterial Communities

**FIGURE 8:**
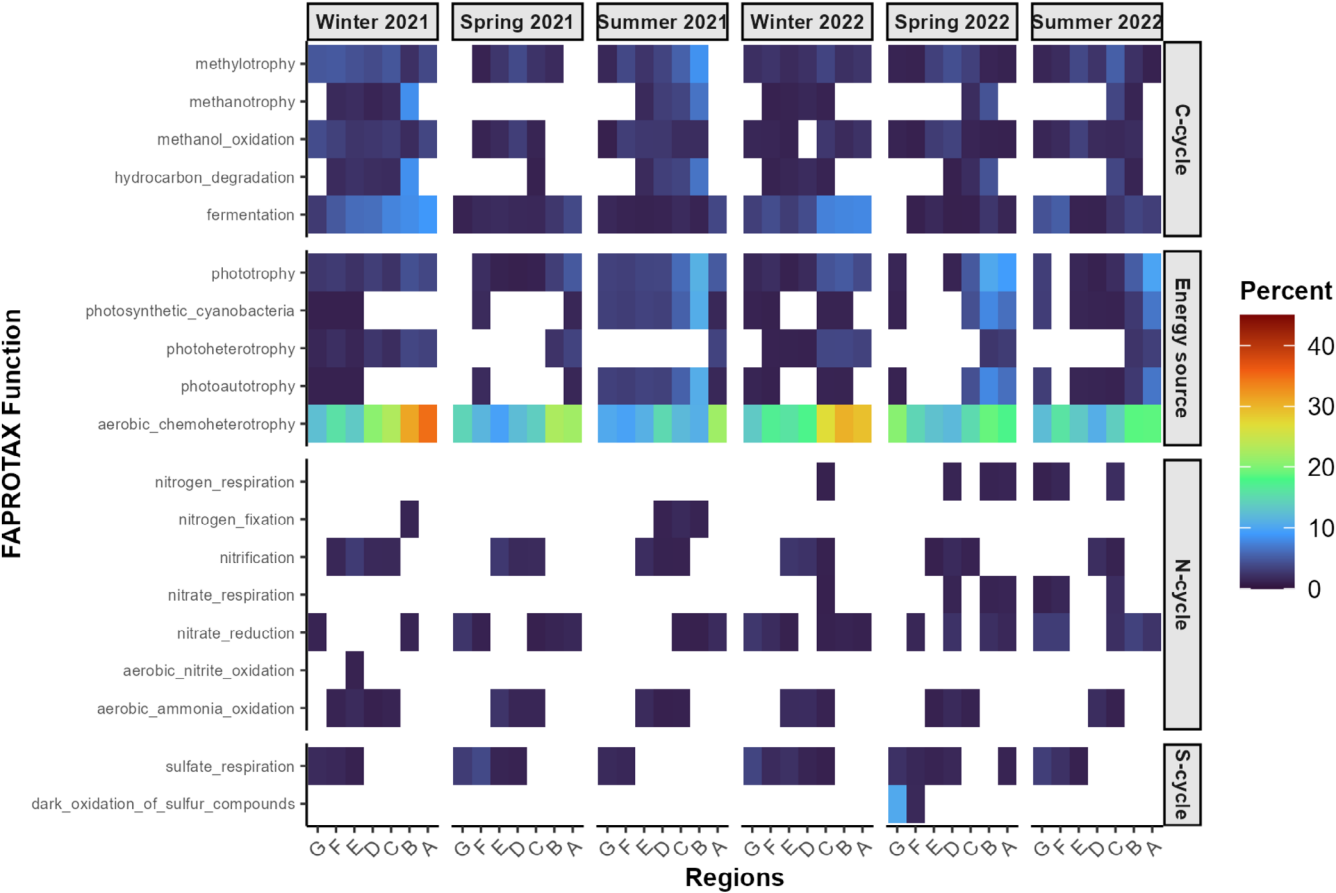
Heatmap of the predicted functions by FAPROTAX normalising the count values to the total ASV read. Only functions with ≥ 1% scaled value are shown. Functions are categorised according to biogeochemical cycles (C‐cycle, N‐cycle, S‐cycle, and Energy source) and sorted by season and region.

Functional prediction based on the FAPROTAX database identified 69 bacterial functional groups, covering 39% of all ASVs in surface water samples, reflecting only a subset of the bacterial community from the Elbe Estuary. Across all regions and seasons, aerobic chemoheterotrophy and fermentation were the dominant predicted functions, consistently showing high relative percentages (Figure 8). Functions associated with phototrophy including photoautotrophy, photosynthetic cyanobacteria, and photoheterotrophy were detected in spring and summer samples, with highest relative percentage observed in summer 2021. These functions were mostly absent in winter. Methylotrophic functions, such as methanol oxidation and methylotrophy, were present at low levels across most samples, with no clear seasonal or spatial trend. While functions related to the N‐cycle were ubiquitous, bacteria involved in nitrification for instance were relatively more abundant in the middle stretch of the estuary and more so in winter, and nitrate reduction especially in summer 2022. A notable spatial and seasonal anomaly was the detection of dark sulfur oxidation, which was present exclusively in samples from spring 2022 and localised primarily in North Sea regions. As these predictions cover only a subset of the bacterial community, future studies using metagenomics, qPCR, or incubation experiments will be helpful for their further validation.

## 4 Discussion

Our study is the first to depict the bacterial diversity within the Elbe Estuary at a detailed level of spatial and temporal scales. The bacterial community in the Elbe Estuary is shaped by multiple factors, including differences between regions, salinity, and seasonality. Strong differences in community structure were observed between the North Sea region and the upper estuary region, while seasonal differences were highest in the upper estuary. Broadly, community composition during winter was more similar to each other in comparison to spring and summer samples that differed more between and within season.

### 4.1 Salinity, Seasonality, and Particles Jointly Shape Microbial Diversity in the Elbe Estuary

While the overall community structure remained relatively stable between the two consecutive years, significant differences in diversity were observed between seasons and along with the salinity gradient. Salinity emerged as a major factor influencing alpha diversity, consistent with previous findings that natural salinity gradients shape bacterial community composition and diversity in estuarine environments (Liu et al. 2015).

Another controlling driver is seasonality. Strong and recurring seasonal shifts in alpha diversity underscore the dominant role of temporal dynamics that shape microbial communities across the estuary. We observed that microbial diversity and richness decrease from winter to spring and summer. This seasonal pattern contrasts with other studies that reported higher microbial diversity during spring and summer, attributed to increased allochthonous inputs during these warmer months (Fang et al. 2023; Laperriere et al. 2020). In the Elbe Estuary, however, allochthonous inputs of nutrients and organic matter are notably higher in winter compared to spring and summer, primarily due to increased river discharge and elevated nitrate concentrations in the incoming river (Dähnke et al. 2008; Schulz, van Beusekom, et al. 2023). Intensified runoff thus contributes to enhanced nutrient availability and greater habitat heterogeneity, as reflected in steeper salinity gradients and elevated levels of suspended particulate matter (Rewrie et al. 2025). In contrast, lower discharge during spring and summer leads to reduced nutrient delivery to the estuary and weaker physicochemical gradients.

This seasonal hydrological dynamic aligns with the pulse‐shunt concept, which posits that high‐flow events in riverine systems promote downstream transport of organic matter and nutrients while limiting in‐stream transformation processes (Raymond et al. 2016). Under high‐flow conditions, water residence time is short relative to microbial generation time, which limits local microbial growth while promoting downstream mixing and the co‐occurrence of diverse microbial communities sourced from upstream or terrestrial habitats (Mueller and Lennon 2025). Mansfeldt et al. 2019 demonstrated that microbial residence time controls both taxonomic composition and functional profiles of communities; short residence times favour fast growing, generalist taxa and increase population mixing, whereas longer residence times allow slow growing, functionally diverse taxa to establish. Consistent with these hydrological and residence time effects, the alpha diversity during spring and summer was remarkably similar in both years. We speculate that this limited seasonal variability may be attributed to continuous mixing of the water column in the Elbe Estuary in summer (Pein et al. 2021), which likely homogenises environmental conditions and reduces spatial heterogeneity in microbial communities (Bittar et al. 2016; Kellogg et al. 2019).

Among the different salinity zones of the Elbe Estuary, the oligohaline region showed notably higher Shannon diversity compared to the other areas. We attribute this to elevated turbidity and suspended particulate matter (SPM) concentrations in the oligohaline MTZ zone, which results from intense resuspension processes and the input of particles of both marine and terrestrial origin (Wolfstein and Kies 1999; Zimmermann 1997; Zimmermann‐Timm 2002). These conditions create more complex habitats that promote greater microbial diversity. Relatively low Chl *a*/SPM ratios in this area (Biederbick et al. 2025) suggest a dominance of non‐living or detrital material over living phytoplankton biomass. The prolonged residence time of particles within the maximum turbidity zone (MTZ) contributes to particle degradation and remineralization (Pein et al. 2021). Bižić‐Ionescu et al. (2015) found that particle‐associated microbial communities exhibit higher diversity than free‐living communities and attributed this to the microscale heterogeneity on and in suspended particles. The complex and dynamic nature of suspended particles offers a variety of ecological niches, potentially fostering microbial diversity. Our findings support the importance of particle degradation (Spieckermann et al. 2022) and the transformation of particulate organic matter (POM) (Dähnke et al. 2022; Kamjunke et al. 2023) in the Elbe Estuary and highlight that biogeochemical interactions on particles are reflected in a diverse microbial community. Thus, the physical dynamics of the MTZ, heterogeneous particle sources, and enhanced residence time support microbial diversity in the Elbe estuary.

Therefore, the Remane concept, originally formulated for macrozoobenthic biodiversity (Remane 1934), describes minimal species richness in transitional waters in estuaries due to physiological stress from intermediate salinities. This pattern was later confirmed for phytoplankton communities in the Elbe Estuary (Muylaert and Sabbe 1999). However, it cannot be applied to bacterial diversity in the Elbe estuary, as well as other estuaries (Crump et al. 2004; Telesh et al. 2011). Instead, our data add to previous investigations of particle‐rich rivers (Lallias et al. 2015), and suggest that bacterial diversity in the oligohaline region is enhanced by high particle load and associated microscale heterogeneity (Bižić‐Ionescu et al. 2015), which effectively counteracts salinity‐related stress predicted by the classical Remane model.

### 4.2 Taxonomic Diversity and Biogeochemical Processes in the Elbe Estuary

The bacterial community composition in the Elbe Estuary reflects patterns commonly observed in estuarine environments worldwide. In total, 36 distinct bacterial phyla were identified, with the most dominant being Proteobacteria, Actinobacteriota, Bacteroidota, Verrucomicrobiota, and Cyanobacteria. These findings are consistent with microbial assemblages reported from anthropogenically impacted aquatic systems such as the Seine estuary (Hervé et al. 2025), the Scheldt (Amadei Martínez et al. 2025), the Grand River in Canada (Virgin et al. 2024), the Dagu River (Ge et al. 2021), and the Yangtze estuary (Xian et al. 2024). Each phylum contributes to essential ecological processes within the estuarine ecosystem, including nutrient cycling, organic matter decomposition, and pollutant degradation.

Among these groups, Proteobacteria emerged as the most abundant and metabolically versatile phylum, with a particularly high presence of *Alphaproteobacteria*, including genera such as *Planktomarina*, *Sulfitobacter*, and *Tabrizicola*. High abundances of *Sulfitobacter* during spring in the North Sea region may reflect its known role in the sulfur cycle, as members of this genus degrade the algal osmolyte DMSP into methanethiol (Ivanova et al. 2004; Luo and Moran 2014).

*Limnohabitans* was the main representative of Gammaproteobacteria in our samples and is an aerobic, heterotrophic bacterium, ubiquitously distributed in freshwater environments. *Limnohabitans* has been identified as an indicator of eutrophic conditions in the Danube River (Fontaine et al. 2023). In winter it was detected even in North Sea samples, while in other seasons it did not reach beyond the MTZ region, which seems to be caused by the higher flow that pushes freshwater communities further into the marine realm before their demise. In contrast, *Rhodoferax*, which showed positive correlations with dissolved oxygen, nitrate, and silicate concentrations, has been proposed as an indicator of good water quality in fluvial systems (Barberoux et al. 2025). In the Elbe Estuary, its occurrence follows a seasonal pattern favouring winter conditions. Its metabolic capabilities include carbon cycling, denitrification, and sulfur oxidation (Finneran et al. 2003; Jin et al. 2020). The unequivocal attribution of indicator species thus appears complicated and highlights variable conditions in the estuary, depending on discharge and nutrient inputs from the catchment and river.

Of the phylum Actinobacteriota, the hgcI clade, CL500‐29 marine group, *Ilumatobacter*, and *Candidatus* Planktophila were predominantly found across the estuary. The hgcl clade is a ubiquitous taxon and typically freshwater inhabitant that is frequently reported in nutrient‐rich rivers and estuarine environments (Li et al. 2017; Liu et al. 2020; Zhang et al. 2014). It is associated with nitrogen‐rich habitats and the utilisation of organic compounds and has the potential to reduce nitrate and phosphate (Ghylin et al. 2014). These functional characteristics, as well as its ability to maintain stable populations across different seasons, could explain its high prevalence in the Elbe River.

In fact, the most absolute amplicon sequence variants (ASVs) in our dataset were affiliated with *Actinobacteria*, particularly an unidentified lineage of the family *Sporichthyaceae* and the CL500‐29 marine group. This underlines their central role in shaping the estuarine microbial community. CL500‐29 marine group, has previously been identified as an indicator of eutrophication in Chinese inland waters (Wang et al. 2024). Its high abundance in the Elbe Estuary suggests a potential link between anthropogenic nutrient inputs and the proliferation of this lineage. This finding reinforces the idea that Actinobacteriota are sensitive indicators of nutrient dynamics in estuarine ecosystems.

Beyond these two major groups, several other phyla also contributed to important ecological functions. Within the phylum Bacteroidota, the genus *Fluviicola* showed a positive correlation with nitrite and ammonium, and a negative correlation with salinity, indicating its preference for freshwater environments. While *Fluviicola* has been proposed as an indicator of good water quality in large river systems such as the Danube (Fontaine et al. 2023), in the Elbe Estuary *Fluviicola* correlated positively with ammonium and nitrite concentrations, raising the question about its reliability as a universal indicator of pristine conditions. Several genera of Bacteroidota were also found to be important players in particle degradation and carbon and nitrogen recycling. *Flavobacterium*, primarily aerobic and chemoorganotrophic, is known for degrading complex biopolymers such as cellulose and pectin (Kirchman 2002). In the Elbe Estuary, *Flavobacterium* abundance correlated positively with nitrate and dissolved oxygen and negatively with salinity, indicating a freshwater preference and greater abundance in colder months, consistent with findings from the Meuse and Mississippi rivers (Barberoux et al. 2025; Payne et al. 2020). Notably, *Pseudarcicella* is known for utilising algal exudates (Kämpfer et al. 2012), supporting its role in post‐bloom organic matter degradation. The genus has been positively correlated with oxygen, silicate, and nitrate, suggesting a preference for nutrient‐rich, oxygenated waters.

In addition to Bacteroidota, Verrucomicrobiota contributed to particle‐associated processes and the breakdown of complex polysaccharides. The genera *Persicirhabdus* and *Terrimicrobium* were among the most abundant. *Persicirhabdus* is also involved in particle degradation. It has been associated with sediment particles and plastic debris (Freitas et al. 2012; Oberbeckmann et al. 2016) and possesses the ability to degrade polysaccharides (Yoon and Lee 2012). Its presence in the estuary may reflect the availability of complex organic substrates and elevated levels of SPM, occurring predominantly in marine‐influenced, particle‐rich regions where it displayed a negative correlation with nitrite (NO_2_^−^) and a strong positive correlation with salinity and pH. *Terrimicrobium*, on the other hand, is considered a potential soil‐derived bacterium. Some species are strictly anaerobic and typically inhabit terrestrial environments (Qiu et al. 2014). In the estuarine context, its occurrence was negatively correlated with dissolved oxygen and positively associated with temperature, indicating that it may be resuspended from sediments into the water column under warmer and more stratified conditions.

*Nitrospira*, a representative genus of the phylum Nitrospirota, plays a key role in nitrification (Daims et al. 2015), a process often driven by degradation of organic matter in the Elbe Estuary (Schulz, Sanders, et al. 2023). Water‐based incubations revealed high nitrification rates in the port (Sanders et al. 2018), and sediments samples from the port of Hamburg exhibited spatially structured ammonia‐ and nitrite‐oxidising communities (Malinowski et al. 2020). In contrast, our data showed that *Nitrospira* reached its highest abundance in the MTZ and was comparatively less abundant in the port area. This suggests that nitrite‐oxidising bacteria (NOB) distribution is strongly influenced by local environmental conditions and habitat type. *Nitrospira* abundance was strongly positively correlated with all matter quality parameters such as suspended particulate matter (SPM), carbon/nitrogen (C/N) ratio, particulate carbon (PC), and particulate nitrogen (PN), while negatively correlated with pH, highlighting their ecological niche in particle‐rich, brackish waters.

An interesting aspect is the occurrence of Cyanobateria, especially *Cyanobium* PCC‐6307, *Planktothrix* NIVA‐CYA 15, and *Pseudanabaena* PCC‐7429, which were predominantly detected during spring and summer. They mostly occurred in the upper estuarine regions, where increased light availability and reduced turbidity promote their proliferation (Soto Ramos et al. 2023). These microorganisms contribute significantly to primary production but may also form harmful algal blooms (HABs) under eutrophic and thermally favourable, that is, warm water, conditions (H. Li et al. 2019). Among these, *Cyanobium* PCC‐6307 is considered a potential producer of cyanotoxins (Jakubowska and Szeląg‐Wasielewska 2015; Leland et al. 2023) and can be considered as an indicator of eutrophic or environmentally degraded water bodies (Barberoux et al. 2025). No relation was observed between Cyanobacteriota abundance and nutrient concentration; *Cyanobium* occurred independently of nitrate depletion in the estuary. Nevertheless, *Cyanobium* lacks the *nifH* gene and is therefore incapable of biological nitrogen fixation. In contrast, certain filamentous strains of *Pseudanabaena* (Acinas et al. 2009) and strains of *Planktothrix* (Pancrace et al. 2017) do possess the *nifH* gene and are considered potential diazotrophs. Therefore, the possibility of N_2_ fixation in the eutrophic estuary cannot be ruled out. Interestingly, the highest frequency of *Pseudanabaena* and *Planktothrix* was consistent with the results of FAPROTAX, which indicated N_2_ fixation in the limnic estuary in spring and summer 2021.

### 4.3 Drivers of Microbial Community Structure and Function in the Elbe Estuary

Besides characterising taxonomic diversity, we also aimed to identify the key environmental drivers that shape microbial community structure in the Elbe Estuary. In many estuaries, community composition appears to be driven by salinity and discharge (Amadei Martínez et al. 2025; Crump et al. 2004; Marín‐Vindas et al. 2023). This appears to be true for our study site, where discharge and salinity explained a substantial proportion of the observed beta‐diversity variation. Additionally, temperature clearly differentiated the samples between seasons, in agreement with previous seasonal studies (Bolaños et al. 2021; Hervé et al. 2025; Ward et al. 2017). We also found that concentrations of nutrients such as silicate, phosphate, and nitrite were correlated with shifts in community composition, particularly during the spring and summer months when biological activity is typically high. This highlights that nutrient availability shapes bacterioplankton dynamics in anthropogenically influenced estuaries (Jeffries et al. 2016).

Complementing these structural insights from beta‐diversity analyses, *putative* functional predictions based on the FAPROTAX database suggest a diverse metabolic potential within the microbial communities of the Elbe Estuary. However, these inferences rely on taxonomic assignments and cover only 39% of the detected ASVs and should therefore be interpreted with caution. Aerobic chemoheterotrophy and fermentation appeared as dominant predicted functions across seasons and regions, underscoring the central role of heterotrophic metabolism in organic matter degradation (Newton et al. 2011) and reflecting the high proportions of Bacteriodota in the samples. Phototrophic functions, including photoautotrophy and photosynthetic cyanobacteria, were seasonally restricted to spring and summer, with peak activity in summer, likely driven by increased light availability (Bunse and Pinhassi 2017; Fortunato et al. 2012). Methanotrophy was present, especially in regions B and C, that is, the port area. *Cand.* Methylopumilus and *Methylotenera* were the main methanotrophs, present in all samples from 2021, indicating a steady but limited contribution to carbon cycling in the estuarine system (Hackbusch et al. 2019). This pattern is consistent with observations that such communities are widespread but environmentally constrained in estuarine sediments (Moussard et al. 2009; Sherry et al. 2016). Also, hydrocarbon degradation functions were found in higher proportions in the port area, which we attributed to the higher pollution with petroleum hydrocarbons in this area (Wetzel et al. 2013).

Nitrogen cycling functions such as nitrate reduction, nitrification, and aerobic ammonia oxidation were ubiquitous, highlighting the continual importance of nitrogen transformations in estuarine biogeochemical processes (Damashek and Francis 2018; Dong et al. 2009; Wankel et al. 2011). Of particular note was the spatially and temporally restricted occurrence of dark sulfur oxidation in estuary samples during spring 2022, suggesting episodic sulfur cycling potentially driven by variable redox conditions, organic matter inputs, or saltwater intrusion, consistent with sulfur dynamics documented in other estuaries (Wasmund et al. 2017). Overall, these results emphasise the complex interplay between environmental gradients and functional microbial processes that shape estuarine microbial ecosystems.

## 5 Conclusion

This study provides the first high‐resolution spatio‐temporal characterisation of bacterial diversity and functional potential in the Elbe Estuary, revealing a complex interplay of environmental drivers. Across two consecutive years, particularly seasonal and discharge‐related changes proved to be stronger determinants of community structure than spatial gradients alone. Salinity emerged as the primary environmental factor shaping microbial assemblages, followed by river discharge and temperature, with nutrient availability modulating community shifts during biologically active periods. Our results demonstrate that particle‐rich, oligohaline regions can sustain high microbial diversity. Functional predictions indicated that heterotrophic metabolism dominates year‐round, while phototrophy, nitrogen fixation, and sulfur oxidation occur more episodically, reflecting seasonal light regimes, nutrient availability, and redox conditions.

We linked microbial diversity to physical and biogeochemical processes and could show that the biogeochemically diverse nature of anthropogenically influenced estuaries is reflected in a diverse and dynamic microbial community. Therefore, our data contribute to microbial ecology assessments and further suggest that the diverse estuarine community will swiftly respond to future changes in hydrology, nutrient loading, and climate.

## Author Contributions

**Vanessa Russnak:** conceptualization, Data curation, Formal analysis, Investigation, Methodology, Visualization, Writing – original draft, Writing – review and editing; **Raphael Koll:** formal analysis, Data curation, Validation, Writing – review and editing; **Sabine Keuter:** formal analysis, Writing – review and editing; **Tina Sanders:** conceptualization, Formal analysis, Methodology, Supervision, Writing – review and editing; **Kirstin Dähnke:** conceptualization, Funding acquisition, Methodology, Supervision, Writing – review and editing.

## Funding

This work was supported by Bundesministerium für Bildung und Forschung, 03F0864C; Deutsche Forschungsgemeinschaft, 407270017/RTG2530, 496691966/FA 1568; Helmholtz Association, MOSES; European Commission; 101000518.

## Ethics Statement

The conducted study did not include work on humans or animals.

## Conflicts of Interest

The authors declare no conflicts of interest.

## Supporting information


**Figure S1:** Bacterial ASV accumulation over number of samples for (A) unfiltered data and (B) 0.001% overall abundance filtered data. (C) depicts the number of ASVs, percentage zeros and total abundance of counts in the individual datasets along different abundance filter levels.
**Figure S2:** Comparison of alpha‐diversity metrics calculated using two rarefaction approaches. Scatterplots show Observed richness, Shannon diversity and Pielou's evenness calculated using single rarefaction (*phyloseq::rarefy_even_depth*; x‐axis) versus the mean of 1000 independent rarefactions (*vegan::rrarefy*; y‐axis). The strong agreement between methods (Spearman *ρ* > 0.99) indicates that alpha‐diversity estimates are robust to stochastic variation introduced by subsampling.
**Figure S3:** Longitudinal profiles of the main environmental parameters along the Elbe Estuary (Elbe‐km 750–600) during 2021 and 2022. Parameters include nitrate (μmol L^−1^), ammonium (μmol L^−1^), nitrite (μmol L^−1^), phosphate (μmol L^−1^), silicate (μmol L^−1^), oxygen (μmol L^−1^), chlorophyll a (μg L^−1^), suspended particulate material, particulars C (mg L^−1^), particulars N (mg L^−1^), C/N ratio and salinity (PSU). Data are grouped by season (winter, spring, and summer) and year. The grey highlighted areas are the Hamburg Port (613–628 km) and the MTZ (651–704 km).
**Figure S4:** Principal coordinates analysis (PCoA) was performed based on the Bray–Curtis distances to visualise the composition of bacterial communities at the (ASV level).
**Table S1:** Pairwise comparisons of Alpha Diversity Indices matrix across environmental categories.
**Table S2:** Results of the permutational multivariate analysis of variance (PERMANOVA) including significant pairwise comparisons between levels of salinity, season, region, and year. The Bray–Curtis dissimilarity was used to calculate distance matrices, and multiple comparisons were corrected using the Benjamini–Hochberg method (FDR).
**Table S3:** Distance‐based redundancy analysis (dbRDA) shows the relationship between physical–chemical and bacterial community. Adjusted R^2^ (%) indicates variance explained by the model, pseudo‐F and *p*‐values (PERMANOVA, *n* = 999) test the significance of explanatory variables.

## Data Availability

The environmental datasets have been submitted to the PANGAEA World Data Center. The 2022 dataset is currently under review and has been assigned a DOI: 10.1594/PANGAEA.979684. The 2021 dataset is also under review and will be assigned a DOI upon publication. For further information, please contact the corresponding author. The 16S rDNA data are archived in the ENA at EMBL‐EBI under accession number PRJEB96293.

## References

[emi470349-bib-0001] Acinas, S. G. , T. H. A. Haverkamp , J. Huisman , and L. J. Stal . 2009. “Phenotypic and Genetic Diversification of *Pseudanabaena* spp. (Cyanobacteria).” ISME Journal 3, no. 1: 31–46. 10.1038/ismej.2008.78. 18769459

[emi470349-bib-0002] Amadei Martínez, L. , K. Sabbe , S. D'hondt , et al. 2025. “Freshwater Discharge and Salinity Drive Taxonomic and Functional Turnover of Microbial Communities in a Turbid Macrotidal Estuary.” Environmental Microbiology Reports 17, no. 4: e70135. 10.1111/1758-2229.70135. 40654031 PMC12256932

[emi470349-bib-0003] Amann, T. , A. Weiss , and J. Hartmann . 2012. “Carbon Dynamics in the Freshwater Part of the Elbe Estuary, Germany: Implications of Improving Water Quality.” Estuarine, Coastal and Shelf Science 107: 112–121. 10.1016/j.ecss.2012.05.012.

[emi470349-bib-0004] Barberoux, V. , A. Anzil , L. Meinertzhagen , T. Nguyen‐Dinh , P. Servais , and I. F. George . 2025. “Spatio‐Temporal Dynamics of Bacterial Community Composition in a Western European Watershed, the Meuse River Watershed.” FEMS Microbiology Ecology 101, no. 4: fiaf022. 10.1093/femsec/fiaf022. 40042978 PMC11916896

[emi470349-bib-0005] Bianchi, T. S. 2006. Biogeochemistry of Estuaries. Oxford University Press. 10.1093/oso/9780195160826.001.0001.

[emi470349-bib-0006] Biederbick, J. , C. Möllmann , E. Hauten , et al. 2025. “Spatial and Temporal Patterns of Zooplankton Trophic Interactions and Carbon Sources in the Eutrophic Elbe Estuary (Germany).” ICES Journal of Marine Science 82, no. 5: fsae189. 10.1093/icesjms/fsae189.

[emi470349-bib-0007] Bittar, T. B. , S. A. Berger , L. M. Birsa , et al. 2016. “Seasonal Dynamics of Dissolved, Particulate and Microbial Components of a Tidal Saltmarsh‐Dominated Estuary Under Contrasting Levels of Freshwater Discharge.” Estuarine, Coastal and Shelf Science 182: 72–85. 10.1016/j.ecss.2016.08.046.

[emi470349-bib-0008] Bižić‐Ionescu, M. , M. Zeder , D. Ionescu , et al. 2015. “Comparison of Bacterial Communities on Limnic Versus Coastal Marine Particles Reveals Profound Differences in Colonization.” Environmental Microbiology 17, no. 10: 3500–3514. 10.1111/1462-2920.12466. 24674021

[emi470349-bib-0009] Boehlich, M. J. , and T. Strotmann . 2008. “The Elbe Estuary.” Die Küste 74: 288–306. https://hdl.handle.net/20.500.11970/101612.

[emi470349-bib-0010] Boehlich, M. J. , and T. Strotmann . 2019. “Das Elbeastuar.” Die Kuste 2019, no. 87: 319–341. 10.18171/1.087106.

[emi470349-bib-0011] Bolaños, L. M. , C. J. Choi , A. Z. Worden , N. Baetge , C. A. Carlson , and S. Giovannoni . 2021. “Seasonality of the Microbial Community Composition in the North Atlantic.” Frontiers in Marine Science 8: 624164. 10.3389/fmars.2021.624164.

[emi470349-bib-0012] Bunse, C. , and J. Pinhassi . 2017. “Marine Bacterioplankton Seasonal Succession Dynamics.” Trends in Microbiology 25, no. 6: 494–505. 10.1016/j.tim.2016.12.013. 28108182

[emi470349-bib-0013] Callahan, B. J. , P. J. McMurdie , M. J. Rosen , A. W. Han , A. J. A. Johnson , and S. P. Holmes . 2016. “DADA2: High‐Resolution Sample Inference From Illumina Amplicon Data.” Nature Methods 13, no. 7: 581–583. 10.1038/nmeth.3869. 27214047 PMC4927377

[emi470349-bib-0014] Caporaso, J. G. , C. L. Lauber , W. A. Walters , et al. 2011. “Global Patterns of 16S rRNA Diversity at a Depth of Millions of Sequences Per Sample.” Proceedings of the National Academy of Sciences of The United States of America 108, no. Suppl 1: 4516–4522. 10.1073/pnas.1000080107. 20534432 PMC3063599

[emi470349-bib-0015] Cloern, J. E. , A. D. Jassby , T. S. Schraga , E. Nejad , and C. Martin . 2017. “Ecosystem Variability Along the Estuarine Salinity Gradient: Examples From Long‐Term Study of San Francisco Bay.” Limnology and Oceanography 62, no. S1: S272–S291. 10.1002/lno.10537.

[emi470349-bib-0016] Crump, B. C. , C. S. Hopkinson , M. L. Sogin , and J. E. Hobbie . 2004. “Microbial Biogeography Along an Estuarine Salinity Gradient: Combined Influences of Bacterial Growth and Residence Time.” Applied and Environmental Microbiology 70, no. 3: 1494–1505. 10.1128/AEM.70.3.1494-1505.2004. 15006771 PMC365029

[emi470349-bib-0017] Dähnke, K. , E. Bahlmann , and K. Emeis . 2008. “A Nitrate Sink in Estuaries? An Assessment by Means of Stable Nitrate Isotopes in the Elbe Estuary.” Limnology and Oceanography 53, no. 4: 1504–1511. 10.4319/lo.2008.53.4.1504.

[emi470349-bib-0018] Dähnke, K. , T. Sanders , Y. Voynova , and S. D. Wankel . 2022. “Nitrogen Isotopes Reveal a Particulate‐Matter‐Driven Biogeochemical Reactor in a Temperate Estuary.” Biogeosciences 19, no. 24: 5879–5891. 10.5194/bg-19-5879-2022.

[emi470349-bib-0019] Daims, H. , E. V. Lebedeva , P. Pjevac , et al. 2015. “Complete Nitrification by Nitrospira Bacteria.” Nature 528, no. 7583: 504–509. 10.1038/nature16461. 26610024 PMC5152751

[emi470349-bib-0020] Damashek, J. , and C. A. Francis . 2018. “Microbial Nitrogen Cycling in Estuaries: From Genes to Ecosystem Processes.” Estuaries and Coasts 41, no. 3: 626–660. 10.1007/s12237-017-0306-2.

[emi470349-bib-0021] Day, J. W. , B. C. Crump , W. M. Kemp , and A. Yáñez‐Arancibia . 2012. Estuarine Ecology. Wiley. 10.1002/9781118412787.

[emi470349-bib-0022] Dong, L. F. , C. J. Smith , S. Papaspyrou , A. Stott , A. M. Osborn , and D. B. Nedwell . 2009. “Changes in Benthic Denitrification, Nitrate Ammonification, and Anammox Process Rates and Nitrate and Nitrite Reductase Gene Abundances Along an Estuarine Nutrient Gradient (The Colne Estuary, United Kingdom).” Applied and Environmental Microbiology 75, no. 10: 3171–3179. 10.1128/AEM.02511-08. 19304834 PMC2681644

[emi470349-bib-0023] Elliott, M. , and D. S. McLusky . 2002. “The Need for Definitions in Understanding Estuaries.” Estuarine, Coastal and Shelf Science 55, no. 6: 815–827. 10.1006/ecss.2002.1031.

[emi470349-bib-0024] Fang, W. , T. Fan , S. Wang , et al. 2023. “Seasonal Changes Driving Shifts in Microbial Community Assembly and Species Coexistence in an Urban River.” Science of the Total Environment 905: 167027. 10.1016/j.scitotenv.2023.167027. 37717779

[emi470349-bib-0025] Finneran, K. T. , C. V. Johnsen , and D. R. Lovley . 2003. “ *Rhodoferax ferrireducens* Sp. Nov., a Psychrotolerant, Facultatively Anaerobic Bacterium That Oxidizes Acetate With the Reduction of Fe(III).” International Journal of Systematic and Evolutionary Microbiology 53, no. 3: 669–673. 10.1099/ijs.0.02298-0. 12807184

[emi470349-bib-0026] Fontaine, L. , L. Pin , D. Savio , et al. 2023. “Bacterial Bioindicators Enable Biological Status Classification Along the Continental Danube River.” Communications Biology 6, no. 1: 862. 10.1038/s42003-023-05237-8. 37596339 PMC10439154

[emi470349-bib-0027] Fortunato, C. S. , and B. C. Crump . 2011. “Bacterioplankton Community Variation Across River to Ocean Environmental Gradients.” Microbial Ecology 62, no. 2: 374–382. 10.1007/s00248-011-9805-z. 21286702

[emi470349-bib-0028] Fortunato, C. S. , L. Herfort , P. Zuber , A. M. Baptista , and B. C. Crump . 2012. “Spatial Variability Overwhelms Seasonal Patterns in Bacterioplankton Communities Across a River to Ocean Gradient.” ISME Journal 6, no. 3: 554–563. 10.1038/ismej.2011.135. 22011718 PMC3280145

[emi470349-bib-0029] Freitas, S. , S. Hatosy , J. A. Fuhrman , et al. 2012. “Global Distribution and Diversity of Marine Verrucomicrobia.” ISME Journal 6, no. 8: 1499–1505. 10.1038/ismej.2012.3. 22318305 PMC3400412

[emi470349-bib-0030] Ge, Y. , Y. Lou , M. Xu , et al. 2021. “Spatial Distribution and Influencing Factors on the Variation of Bacterial Communities in an Urban River Sediment.” Environmental Pollution 272: 115984. 10.1016/j.envpol.2020.115984. 33168378

[emi470349-bib-0031] Geerts, L. , K. Wolfstein , S. Jacobs , S. Van Damme , and W. Vandenbruwaene . 2012. Zonation of the TIDE Estuaries .

[emi470349-bib-0032] Ghylin, T. W. , S. L. Garcia , F. Moya , et al. 2014. “Comparative Single‐Cell Genomics Reveals Potential Ecological Niches for the Freshwater acI Actinobacteria Lineage.” ISME Journal 8, no. 12: 2503–2516. 10.1038/ismej.2014.135. 25093637 PMC4260696

[emi470349-bib-0033] Hackbusch, S. , A. Wichels , and I. Bussmann . 2019. “Abundance, Activity and Diversity of Methanotrophic Bacteria in the Elbe Estuary and Southern North Sea.” Aquatic Microbial Ecology 83, no. 1: 35–48. 10.3354/ame01899.

[emi470349-bib-0034] Hall, E. K. , E. S. Bernhardt , R. L. Bier , et al. 2018. “Understanding How Microbiomes Influence the Systems They Inhabit.” Nature Microbiology 3, no. 9: 977–982. 10.1038/s41564-018-0201-z. 30143799

[emi470349-bib-0035] Hansen, H. P. , and F. Koroleff . 2007. “Determination of Nutrients.” In Methods of Seawater Analysis: Third, Completely Revised and Extended Edition, 159–228. Wiley Blackwell. 10.1002/9783527613984.ch10.

[emi470349-bib-0036] Harrell, F. E. 2025. Hmisc: Harrell Miscellaneous (Version 5.2–3). CRAN. https://CRAN.R‐Project.Org/Package=Hmisc.

[emi470349-bib-0037] Hervé, V. , J. Morelle , J. Lambourdière , P. J. Lopez , and P. Claquin . 2025. “Together Throughout the Year: Seasonal Patterns of Bacterial and Eukaryotic Microbial Communities in a Macrotidal Estuary.” Environmental Microbiomes 20, no. 1: 8. 10.1186/s40793-025-00664-y. PMC11748528 39833892

[emi470349-bib-0038] Hoitink, A. J. F. , and D. A. Jay . 2016. “Tidal River Dynamics: Implications for Deltas.” Reviews of Geophysics 54, no. 1: 240–272. 10.1002/2015RG000507.

[emi470349-bib-0039] Ivanova, E. P. , N. M. Gorshkova , T. Sawabe , et al. 2004. “ *Sulfitobacter delicatus* Sp. Nov. and *Sulfitobacter dubius* sp. Nov., Respectively From a Starfish ( *Stellaster equestris* ) and Sea Grass ( *Zostera marina* ).” International Journal of Systematic and Evolutionary Microbiology 54, no. 2: 475–480. 10.1099/ijs.0.02654-0. 15023963

[emi470349-bib-0040] Jakubowska, N. , and E. Szeląg‐Wasielewska . 2015. “Toxic Picoplanktonic Cyanobacteria—Review.” Marine Drugs 13, no. 3: 1497–1518. 10.3390/md13031497. 25793428 PMC4377996

[emi470349-bib-0041] Jeffrey, S. W. , and G. F. Humphrey . 1975. “New Spectrophotometric Equations for Determining Chlorophylls a, b, c1 and c2 in Higher Plants, Algae and Natural Phytoplankton.” Biochemie und Physiologie der Pflanzen 167, no. 2: 191–194. 10.1016/S0015-3796(17)30778-3.

[emi470349-bib-0042] Jeffries, T. C. , M. L. Schmitz Fontes , D. P. Harrison , et al. 2016. “Bacterioplankton Dynamics Within a Large Anthropogenically Impacted Urban Estuary.” Frontiers in Microbiology 6: 1438. 10.3389/fmicb.2015.01438. 26858690 PMC4726783

[emi470349-bib-0043] Jin, C. Z. , Y. Zhuo , X. Wu , et al. 2020. “Genomic and Metabolic Insights Into Denitrification, Sulfur Oxidation, and Multidrug Efflux Pump Mechanisms in the Bacterium Rhodoferax Sediminis sp. Nov.” Microorganisms 8, no. 2: 262. 10.3390/microorganisms8020262. 32075304 PMC7074706

[emi470349-bib-0044] Kamjunke, N. , H. Brix , G. Flöser , et al. 2023. “Large‐Scale Nutrient and Carbon Dynamics Along the River‐Estuary‐Ocean Continuum.” Science of the Total Environment 890: 164421. 10.1016/j.scitotenv.2023.164421. 37244620

[emi470349-bib-0045] Kämpfer, P. , H. J. Busse , I. Longaric , R. Rosselló‐Móra , H. Galatis , and N. Lodders . 2012. “ *Pseudarcicella Hirudinis* Gen. Nov., sp. Nov., Isolated From the Skin of the Medical Leech *Hirudo medicinalis* .” International Journal of Systematic and Evolutionary Microbiology 62, no. 9: 2247–2251. 10.1099/ijs.0.037390-0. 22081719

[emi470349-bib-0046] Kang, I. , S. Kim , M. R. Islam , and J. C. Cho . 2017. “The First Complete Genome Sequences of the acI Lineage, the Most Abundant Freshwater Actinobacteria, Obtained by Whole‐Genome‐Amplification of Dilution‐To‐Extinction Cultures.” Scientific Reports 7: 42252. 10.1038/srep42252. 28186143 PMC5301498

[emi470349-bib-0047] Kellogg, C. T. E. , J. W. McClelland , K. H. Dunton , and B. C. Crump . 2019. “Strong Seasonality in Arctic Estuarine Microbial Food Webs.” Frontiers in Microbiology 10: 2628. 10.3389/fmicb.2019.02628. 31849850 PMC6896822

[emi470349-bib-0048] Kennish, M. J. 2002. “Environmental Threats and Environmental Future of Estuaries.” Environmental Conservation 29, no. 1: 78–107. 10.1017/S0376892902000061.

[emi470349-bib-0049] Kirchman, D. L. 2002. “The Ecology of Cytophaga‐Flavobacteria in Aquatic Environments.” FEMS Microbiology Ecology 39, no. 2: 91–100. 10.1111/j.1574-6941.2002.tb00910.x. 19709188

[emi470349-bib-0050] Kirchman, D. L. , A. I. Dittel , R. R. Malmstrom , and M. T. Cottrell . 2005. “Biogeography of Major Bacterial Groups in the Delaware Estuary.” Limnology and Oceanography 50, no. 5: 1697–1706. 10.4319/lo.2005.50.5.1697.

[emi470349-bib-0051] Kozich, J. J. , S. L. Westcott , N. T. Baxter , S. K. Highlander , and P. D. Schloss . 2013. “Development of a Dual‐Index Sequencing Strategy and Curation Pipeline for Analyzing Amplicon Sequence Data on the Miseq Illumina Sequencing Platform.” Applied and Environmental Microbiology 79, no. 17: 5112–5120. 10.1128/AEM.01043-13. 23793624 PMC3753973

[emi470349-bib-0052] Krueger, F. 2015. “Trim Galore!: A Wrapper Around Cutadapt and Fast QC to Consistently Apply Adapter and Quality Trimming to Fast Q Files, With Extra Functionality for RRBS Data.” Babraham Institute 26, no. 7: 530–540.

[emi470349-bib-0053] Lallias, D. , J. G. Hiddink , V. G. Fonseca , et al. 2015. “Environmental Metabarcoding Reveals Heterogeneous Drivers of Microbial Eukaryote Diversity in Contrasting Estuarine Ecosystems.” ISME Journal 9, no. 5: 1208–1221. 10.1038/ismej.2014.213. 25423027 PMC4409164

[emi470349-bib-0054] Laperriere, S. M. , R. H. Hilderbrand , S. R. Keller , R. Trott , and A. E. Santoro . 2020. “Headwater Stream Microbial Diversity and Function Across Agricultural and Urban Land Use Gradients.” Applied and Environmental Microbiology 86, no. 11: e00018‐20. 10.1128/AEM.00018-20. 32245755 PMC7237783

[emi470349-bib-0055] Leland, N. J. , K. C. Pearson , M. K. Burke , J. T. Miller , A. Watts , and J. F. Haney . 2023. “Isolation of Picocyanobacteria (Order Synechococcales) and Occurrence of the Cyanotoxin Anatoxin‐A in a Shallow Mesotrophic Pond.” Journal of Water Resource and Protection 15, no. 6: 299–314. 10.4236/jwarp.2023.156017.

[emi470349-bib-0056] Li, H. , A. Alsanea , M. Barber , and R. Goel . 2019. “High‐Throughput DNA Sequencing Reveals the Dominance of Pico‐ and Other Filamentous Cyanobacteria in an Urban Freshwater Lake.” Science of the Total Environment 661: 465–480. 10.1016/j.scitotenv.2019.01.141. 30677691

[emi470349-bib-0057] Li, J. , X. Jiang , Z. Jing , et al. 2017. “Spatial and Seasonal Distributions of Bacterioplankton in the Pearl River Estuary: The Combined Effects of Riverine Inputs, Temperature, and Phytoplankton.” Marine Pollution Bulletin 125, no. 1–2: 199–207. 10.1016/j.marpolbul.2017.08.026. 28823423

[emi470349-bib-0058] Liu, J. , B. Fu , H. Yang , M. Zhao , B. He , and X. H. Zhang . 2015. “Phylogenetic Shifts of Bacterioplankton Community Composition Along the Pearl Estuary: The Potential Impact of Hypoxia and Nutrients.” Frontiers in Microbiology 6: 64. 10.3389/fmicb.2015.00064. 25713564 PMC4322608

[emi470349-bib-0059] Liu, Q. , Y. Zhang , H. Wu , et al. 2020. “A Review and Perspective of eDNA Application to Eutrophication and HAB Control in Freshwater and Marine Ecosystems.” Microorganisms 8, no. 3: 417. 10.3390/microorganisms8030417. 32188048 PMC7143994

[emi470349-bib-0060] Louca, S. , L. W. Parfrey , and M. Doebeli . 2016. “Decoupling Function and Taxonomy in the Global Ocean Microbiome.” Science 353, no. 6305: 1272–1277. 10.1126/science.aaf4507. 27634532

[emi470349-bib-0061] Luo, H. , and M. A. Moran . 2014. “Evolutionary Ecology of the Marine Roseobacter Clade.” Microbiology and Molecular Biology Reviews 78, no. 4: 573–587. 10.1128/mmbr.00020-14. 25428935 PMC4248658

[emi470349-bib-0062] Malinowski, M. , M. Alawi , I. Krohn , et al. 2020. “Deep amoA Amplicon Sequencing Reveals Community Partitioning Within Ammonia‐Oxidizing Bacteria in the Environmentally Dynamic Estuary of the River Elbe.” Scientific Reports 10, no. 1: 17165. 10.1038/s41598-020-74163-0. 33051504 PMC7555866

[emi470349-bib-0063] Mansfeldt, C. , S. Achermann , Y. Men , et al. 2019. “Microbial Residence Time Is a Controlling Parameter of the Taxonomic Composition and Functional Profile of Microbial Communities.” ISME Journal 13, no. 6: 1589–1601. 10.1038/s41396-019-0371-6. 30787397 PMC6544533

[emi470349-bib-0064] Marín‐Vindas, C. , M. Sebastián , C. Ruiz‐González , V. Balagué , L. Vega‐Corrales , and J. M. Gasol . 2023. “Shifts in Bacterioplankton Community Structure Between Dry and Wet Seasons in a Tropical Estuary Strongly Affected by Riverine Discharge.” Science of the Total Environment 903: 166104. 10.1016/j.scitotenv.2023.166104. 37558065

[emi470349-bib-0065] Martens, N. , V. Russnak , J. Woodhouse , H. P. Grossart , and C. E. Schaum . 2024. “Metabarcoding Reveals Potentially Mixotrophic Flagellates and Picophytoplankton as Key Groups of Phytoplankton in the Elbe Estuary.” Environmental Research 252: 119126. 10.1016/j.envres.2024.119126. 38734293

[emi470349-bib-0066] Martin, M. 2011. “Cutadapt Removes Adapter Sequences From High‐Throughput Sequencing Reads.” EMBnet.Journal 17, no. 1: 10. 10.14806/ej.17.1.200.

[emi470349-bib-0067] McMurdie, P. J. , and S. Holmes . 2011. “Phyloseq: A Bioconductor Package for Handling and Analysis of High‐Throughput Phylogenetic Sequence Data.” Biocomputing 2012: 235–246. 10.1142/9789814366496_0023. PMC3357092 22174279

[emi470349-bib-0068] Moftakhari, H. R. , D. A. Jay , S. A. Talke , T. Kukulka , and P. D. Bromirski . 2013. “A Novel Approach to Flow Estimation in Tidal Rivers.” Water Resources Research 49, no. 8: 4817–4832. 10.1002/wrcr.20363.

[emi470349-bib-0069] Moussard, H. , N. Stralis‐Pavese , L. Bodrossy , J. D. Neufeld , and J. Colin Murrell . 2009. “Identification of Active Methylotrophic Bacteria Inhabiting Surface Sediment of a Marine Estuary.” Environmental Microbiology Reports 1, no. 5: 424–433. 10.1111/j.1758-2229.2009.00063.x. 23765896

[emi470349-bib-0070] Mueller, E. A. , and J. T. Lennon . 2025. “Residence Time Structures Microbial Communities Through Niche Partitioning.” Ecology Letters 28, no. 2: e70093. 10.1111/ele.70093. 40007481 PMC11862987

[emi470349-bib-0071] Muylaert, K. , and K. Sabbe . 1999. “Spring Phytoplankton Assemblages in and Around the Maximum Turbidity Zone of the Estuaries of the Elbe (Germany), the Schelde (Belgium/The Netherlands) and the Gironde (France).” Journal of Marine Systems 22, no. 2–3: 133–149. 10.1016/S0924-7963(99)00037-8.

[emi470349-bib-0072] Muyzer, G. , and A. G. De Waal Uitierlinden . 1993. “Profiling of Complex Microbial Populations by Denaturing Gradient Gel Electrophoresis Analysis of Polymerase Chain Reaction‐Amplified Genes Coding for 16S rRNA.” Applied and Environmental Microbiology 59, no. 3: 695–700. 10.1128/aem.59.3.695-700.1993. 7683183 PMC202176

[emi470349-bib-0073] Newton, R. J. , S. E. Jones , A. Eiler , K. D. McMahon , and S. Bertilsson . 2011. “A Guide to the Natural History of Freshwater Lake Bacteria.” Microbiology and Molecular Biology Reviews 75, no. 1: 14–49. 10.1128/mmbr.00028-10. 21372319 PMC3063352

[emi470349-bib-0074] Newton, R. J. , S. E. Jones , M. R. Helmus , and K. D. McMahon . 2007. “Phylogenetic Ecology of the Freshwater Actinobacteria acI Lineage.” Applied and Environmental Microbiology 73, no. 22: 7169–7176. 10.1128/AEM.00794-07. 17827330 PMC2168227

[emi470349-bib-0075] Oberbeckmann, S. , A. M. Osborn , and M. B. Duhaime . 2016. “Microbes on a Bottle: Substrate, Season and Geography Influence Community Composition of Microbes Colonizing Marine Plastic Debris.” PLoS One 11, no. 8: e0159289. 10.1371/journal.pone.0159289. 27487037 PMC4972250

[emi470349-bib-0076] O'Connor, J. A. , D. V. Erler , A. Ferguson , and D. T. Maher . 2022. “The Tidal Freshwater River Zone: Physical Properties and Biogeochemical Contribution to Estuarine Hypoxia and Acidification—The Hydrologic Switch.” Estuarine, Coastal and Shelf Science 268: 107786. 10.1016/j.ecss.2022.107786.

[emi470349-bib-0077] Oksanen, J. , G. L. Simpson , F. G. Blanchet , et al. 2022. Vegan: Community Ecology Package (R Package Version 2.6–4).

[emi470349-bib-0078] Pancrace, C. , M. A. Barny , R. Ueoka , et al. 2017. “Insights Into the Planktothrix Genus: Genomic and Metabolic Comparison of Benthic and Planktic Strains.” Scientific Reports 7: 41181. 10.1038/srep41181. 28117406 PMC5259702

[emi470349-bib-0079] Papenmeier, S. , K. Schrottke , and A. Bartholomä . 2014. “Over Time and Space Changing Characteristics of Estuarine Suspended Particles in the German Weser and Elbe Estuaries.” Journal of Sea Research 85: 104–115. 10.1016/j.seares.2013.03.010.

[emi470349-bib-0080] Payne, J. T. , C. R. Jackson , J. J. Millar , and C. A. Ochs . 2020. “Timescales of Variation in Diversity and Production of Bacterioplankton Assemblages in the Lower Mississippi River.” PLoS One 15, no. 4: e0230945. 10.1371/journal.pone.0230945. 32255790 PMC7138331

[emi470349-bib-0081] Pein, J. , A. Eisele , T. Sanders , et al. 2021. “Seasonal Stratification and Biogeochemical Turnover in the Freshwater Reach of a Partially Mixed Dredged Estuary.” Frontiers in Marine Science 8: 623714. 10.3389/fmars.2021.623714.

[emi470349-bib-0082] Petersen, W. , F. Schroeder , and F. D. Bockelmann . 2011. “FerryBox—Application of Continuous Water Quality Observations Along Transects in the North Sea.” Ocean Dynamics 61, no. 10: 1541–1554. 10.1007/s10236-011-0445-0.

[emi470349-bib-0083] Qiu, Y.‐L. , X. Kuang , X. Shi , X. Yuan , and R. Guo . 2014. “Terrimicrobium Sacchariphilum Gen. Nov., sp. Nov., an Anaerobic Bacterium of the Class ‘Spartobacteria’ in the Phylum Verrucomicrobia, Isolated From a Rice Paddy Field.” International Journal of Systematic and Evolutionary Microbiology 64, no. Pt_5: 1718–1723. 10.1099/ijs.0.060244-0. 24535138

[emi470349-bib-0084] Quast, C. , E. Pruesse , P. Yilmaz , et al. 2012. “The SILVA Ribosomal RNA Gene Database Project: Improved Data Processing and Web‐Based Tools.” Nucleic Acids Research 41: D590–D596. 10.1093/nar/gks1219. 23193283 PMC3531112

[emi470349-bib-0085] Raymond, P. A. , J. Hartmann , R. Lauerwald , et al. 2013. “Global Carbon Dioxide Emissions From Inland Waters.” Nature 503, no. 7476: 355–359. 10.1038/nature12760. 24256802

[emi470349-bib-0086] Raymond, P. A. , J. E. Saiers , and W. V. Sobczak . 2016. “Hydrological and Biogeochemical Controls on Watershed Dissolved Organic Matter Transport: Pulse‐ Shunt Concept.” Ecology 97, no. 1: 5–16. 10.1890/14-1684.1. 27008769

[emi470349-bib-0087] Remane, A. 1934. “Die Brackwasserfauna.” Verhandlungen der Deutschen Zoologischen Gesellschaft: 34–74.

[emi470349-bib-0088] Rewrie, L. C. V. , B. Baschek , J. E. E. van Beusekom , A. Körtzinger , G. Ollesch , and Y. G. Voynova . 2023. “Recent Inorganic Carbon Increase in a Temperate Estuary Driven by Water Quality Improvement and Enhanced by Droughts.” Biogeosciences 20, no. 24: 4931–4947. 10.5194/bg-20-4931-2023.

[emi470349-bib-0089] Rewrie, L. C. V. , B. Baschek , J. E. E. van Beusekom , et al. 2025. “Impact of Primary Production and Net Ecosystem Metabolism on Carbon and Nutrient Cycling at the Land‐Sea Interface.” Frontiers in Marine Science 12: 1548463. 10.3389/fmars.2025.1548463.

[emi470349-bib-0090] Sanders, T. , A. Schöl , and K. Dähnke . 2018. “Hot Spots of Nitrification in the Elbe Estuary and Their Impact on Nitrate Regeneration.” Estuaries and Coasts 41, no. 1: 128–138. 10.1007/s12237-017-0264-8.

[emi470349-bib-0091] Schulz, G. , T. Sanders , Y. G. Voynova , H. W. Bange , and K. Dähnke . 2023. “Seasonal Variability of Nitrous Oxide Concentrations and Emissions Along the Elbe Estuary.” Biogeosciences 20: 3229–3247. 10.5194/bg-2023-35.

[emi470349-bib-0092] Schulz, G. , J. E. E. van Beusekom , J. Jacob , et al. 2023. “Low Discharge Intensifies Nitrogen Retention in Rivers—A Case Study in the Elbe River.” Science of the Total Environment 904: 166740. 10.1016/j.scitotenv.2023.166740. 37659520

[emi470349-bib-0093] Sherry, A. , K. A. Osborne , F. R. Sidgwick , N. D. Gray , and H. M. Talbot . 2016. “A Temperate River Estuary Is a Sink for Methanotrophs Adapted to Extremes of pH, Temperature and Salinity.” Environmental Microbiology Reports 8, no. 1: 122–131. 10.1111/1758-2229.12359. 26617278 PMC4959530

[emi470349-bib-0094] Soto Ramos, I. M. , B. Crooke , B. Seegers , I. Cetinić , M. K. Cambazoglu , and B. Armstrong . 2023. “Spatial and Temporal Characterization of Cyanobacteria Blooms in the Mississippi Sound and Their Relationship to the Bonnet Carré Spillway Openings.” Harmful Algae 127: 102472. 10.1016/j.hal.2023.102472. 37544672

[emi470349-bib-0095] Spieckermann, M. , A. Gröngröft , M. Karrasch , A. Neumann , and A. Eschenbach . 2022. “Oxygen Consumption of Resuspended Sediments of the Upper Elbe Estuary: Process Identification and Prognosis.” Aquatic Geochemistry 28, no. 1: 1–25. 10.1007/s10498-021-09401-6.

[emi470349-bib-0096] Telesh, I. , H. Schubert , and S. Skarlato . 2011. “Revisiting Remane's Concept: Evidence for High Plankton Diversity and a Protistan Species Maximum in the Horohalinicum of the Baltic Sea.” Marine Ecology Progress Series 421: 1–11. 10.3354/meps08928.

[emi470349-bib-0097] Tobias‐Hünefeldt, S. P. , J. E. E. van Beusekom , V. Russnak , K. Dähnke , W. R. Streit , and H. P. Grossart . 2024. “Seasonality, Rather Than Estuarine Gradient or Particle Suspension/Sinking Dynamics, Determines Estuarine Carbon Distributions.” Science of the Total Environment 926: 171962. 10.1016/j.scitotenv.2024.171962. 38537819

[emi470349-bib-0098] Virgin, T. L. , P. Sonthiphand , S. Coyotzi , et al. 2024. “Microbial Communities Change Along the 300 Km Length of the Grand River for Extreme High‐and Low‐Flow Regimes.” Canadian Journal of Microbiology 70, no. 7: 289–302. 10.1139/cjm-2023-0092. 38747604

[emi470349-bib-0099] Wang, B. , K. Hu , C. Li , et al. 2024. “Geographic Distribution of Bacterial Communities of Inland Waters in China.” Environmental Research 249: 118337. 10.1016/j.envres.2024.118337. 38325783

[emi470349-bib-0100] Wang, H. , F. Chen , C. Zhang , M. Wang , and J. Kan . 2021. “Estuarine Gradients Dictate Spatiotemporal Variations of Microbiome Networks in the Chesapeake Bay.” Environmental Microbiomes 16, no. 1: 22. 10.1186/s40793-021-00392-z. PMC8627074 34838139

[emi470349-bib-0101] Wankel, S. D. , A. C. Mosier , C. M. Hansel , A. Paytan , and C. A. Francis . 2011. “Spatial Variability in Nitrification Rates and Ammonia‐Oxidizing Microbial Communities in the Agriculturally Impacted Elkhorn Slough Estuary, California.” Applied and Environmental Microbiology 77, no. 1: 269–280. 10.1128/AEM.01318-10. 21057023 PMC3019697

[emi470349-bib-0102] Ward, C. S. , C. M. Yung , K. M. Davis , et al. 2017. “Annual Community Patterns Are Driven by Seasonal Switching Between Closely Related Marine Bacteria.” ISME Journal 11, no. 6: 1412–1422. 10.1038/ismej.2017.4. 28234350 PMC5437356

[emi470349-bib-0103] Wasmund, K. , M. Mußmann , and A. Loy . 2017. “The Life Sulfuric: Microbial Ecology of Sulfur Cycling in Marine Sediments.” Environmental Microbiology Reports 9, no. 4: 323–344. 10.1111/1758-2229.12538. 28419734 PMC5573963

[emi470349-bib-0104] Wetzel, M. A. , D. S. Wahrendorf , and P. C. von der Ohe . 2013. “Sediment Pollution in the Elbe Estuary and Its Potential Toxicity at Different Trophic Levels.” Science of the Total Environment 449: 199–207. 10.1016/j.scitotenv.2013.01.016. 23428749

[emi470349-bib-0105] Wolfstein, K. , and L. Kies . 1999. “Composition of Suspended Particulate Matter in the Elbe Estuary: Implications for Biological and Transportation Processes.” Deutsche Hydrographische Zeitschrift = German Journal of Hydrography 51, no. 4: 453–463. 10.1007/BF02764166.

[emi470349-bib-0106] Xian, W. D. , J. Chen , Z. Zheng , et al. 2024. “Water Masses Influence the Variation of Microbial Communities in the Yangtze River Estuary and Its Adjacent Waters.” Frontiers in Microbiology 15: 1367062. 10.3389/fmicb.2024.1367062. 38572235 PMC10987813

[emi470349-bib-0107] Yoon, J. H. , and S. Y. Lee . 2012. “Winogradskyella Multivorans sp. Nov., a Polysaccharidedegrading Bacterium Isolated From Seawater of an Oyster Farm.” Antonie van Leeuwenhoek International Journal of General and Molecular Microbiology 102, no. 2: 231–238. 10.1007/s10482-012-9729-8. 22476412

[emi470349-bib-0108] Zhang, Y. , Z. Zhao , M. Dai , N. Jiao , and G. J. Herndl . 2014. “Drivers Shaping the Diversity and Biogeography of Total and Active Bacterial Communities in the South China Sea.” Molecular Ecology 23, no. 9: 2260–2274. 10.1111/mec.12739. 24684298 PMC4230472

[emi470349-bib-0109] Zimmermann, H. 1997. “The Microbial Community on Aggregates in the Elbe Estuary, Germany.” Aquatic Microbial Ecology 13, no. 1: 37–46. 10.3354/ame013037.

[emi470349-bib-0110] Zimmermann‐Timm, H. 2002. “Characteristics, Dynamics and Importance of Aggregates in Rivers—An Invited Review.” International Review of Hydrobiology 87, no. 2–3: 197–240. 10.1002/1522-2632(200205)87:2/3<197::AID-IROH197>3.0.CO;2-7.

[emi470349-bib-0111] Zimmermann‐Timm, H. , H. Holst , and S. Muller . 1998. “Seasonal Dynamics of Aggregates and Their Typical Biocoenosis in the Elbe Estuary.” Estuaries 21, no. 4: 613.

