# Supplementary material for: The Elbe Estuary Microbiome Shifts With Salinity and Discharge and Depends on Fresh Organic Matter and Nutrient Availability
Source: Environ Microbiol Rep. 2026 Apr 28;18(3):e70349. doi: 10.1111/1758-2229.70349 (PMC13124656; doi:10.1111/1758-2229.70349)
Supplement: Supplementary file 1 — Figure S1: Bacterial ASV accumulation over number of samples for (A) unfiltered data and (B) 0.001% overall abundance filtered data. (C) depicts the number of ASVs, percentage zeros and total abundance of counts in the individual datasets along different abundance filter levels. Figure S2: Comparison of alpha‐diversity metrics calculated using two rarefaction approaches. Scatterplots show Observed richness, Shannon diversity and Pielou's evenness calculated using single rarefaction (phyloseq::rarefy_even_depth; x‐axis) versus the mean of 1000 independent rarefactions (vegan::rrarefy; y‐axis). The strong agreement between methods (Spearman ρ > 0.99) indicates that alpha‐diversity estimates are robust to stochastic variation introduced by subsampling. Figure S3: Longitudinal profiles of the main environmental parameters along the Elbe Estuary (Elbe‐km 750–600) during 2021 and 2022. Parameters include nitrate (μmol L−1), ammonium (μmol L−1), nitrite (μmol L−1), phosphate (μmol L−1), silicate (μmol L−1), oxygen (μmol L−1), chlorophyll a (μg L−1), suspended particulate material, particulars C (mg L−1), particulars N (mg L−1), C/N ratio and salinity (PSU). Data are grouped by season (winter, spring, and summer) and year. The grey highlighted areas are the Hamburg Port (613–628 km) and the MTZ (651–704 km). Figure S4: Principal coordinates analysis (PCoA) was performed based on the Bray–Curtis distances to visualise the composition of bacterial communities at the (ASV level). Table S1: Pairwise comparisons of Alpha Diversity Indices matrix across environmental categories. Table S2: Results of the permutational multivariate analysis of variance (PERMANOVA) including significant pairwise comparisons between levels of salinity, season, region, and year. The Bray–Curtis dissimilarity was used to calculate distance matrices, and multiple comparisons were corrected using the Benjamini–Hochberg method (FDR). Table S3: Distance‐based redundancy analysis (dbRDA) shows the relations [file EMI4-18-e70349-s001.zip › emi470349-sup-0001-FigureS1-S4-TableS1-S3.docx]

**Supplementary Material**

**The Elbe Estuary microbiome shifts with salinity and discharge and depends on fresh organic matter and nutrient availability**

Vanessa Russnak^1^, Raphael Koll^2^, Sabine Keuter^1^, Tina Sanders^1^, Kirstin Dähnke^1^

^1^ Helmholtz-Centre Hereon, Institute of Carbon Cycles, Max-Planck-Strasse 1, 21502 Geesthacht, Germany

^2^ Institute of Marine Ecosystem and Fishery Science, Universität Hamburg, Grosse Elbstrasse 133, 22767 Hamburg, Germany

**Corresponding author:** Vanessa Russnak


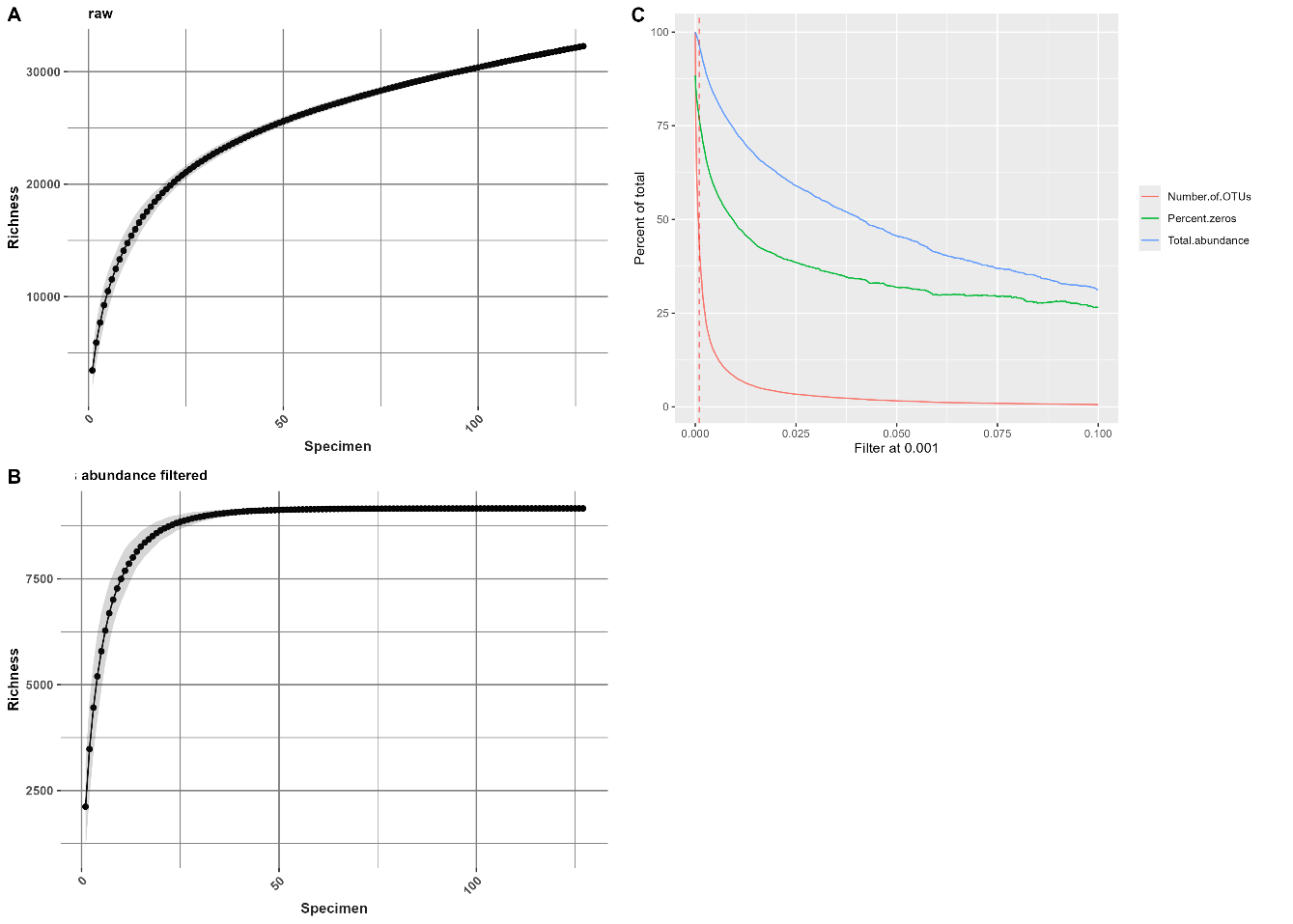


**Figure S1** **|** Bacterial ASV accumulation over number of samples for (A) unfiltered data and (B) 0.001% overall abundance filtered data. (C) depicts the number of ASVs, percentage zeros and total abundance of counts in the individual datasets along different abundance filter levels.

**Methods S1:** **Rarefaction depth and robustness analysis**

Sequencing depths ranged from 5777 to 14756 reads per sample (mean = 10442 ± 2120), rarefication depth was 5777, retaining 127 of 129 samples (99%). Key alpha-diversity patterns were robust to an alternative rarefaction approach using *vegan::rrarefy*. Alpha-diversity estimates obtained from single rarefaction (phyloseq) were highly consistent with the mean of 1,000 independent rarefactions, with Spearman correlations exceeding 0.99 for Observed richness, Shannon diversity, and Pielou’s evenness.


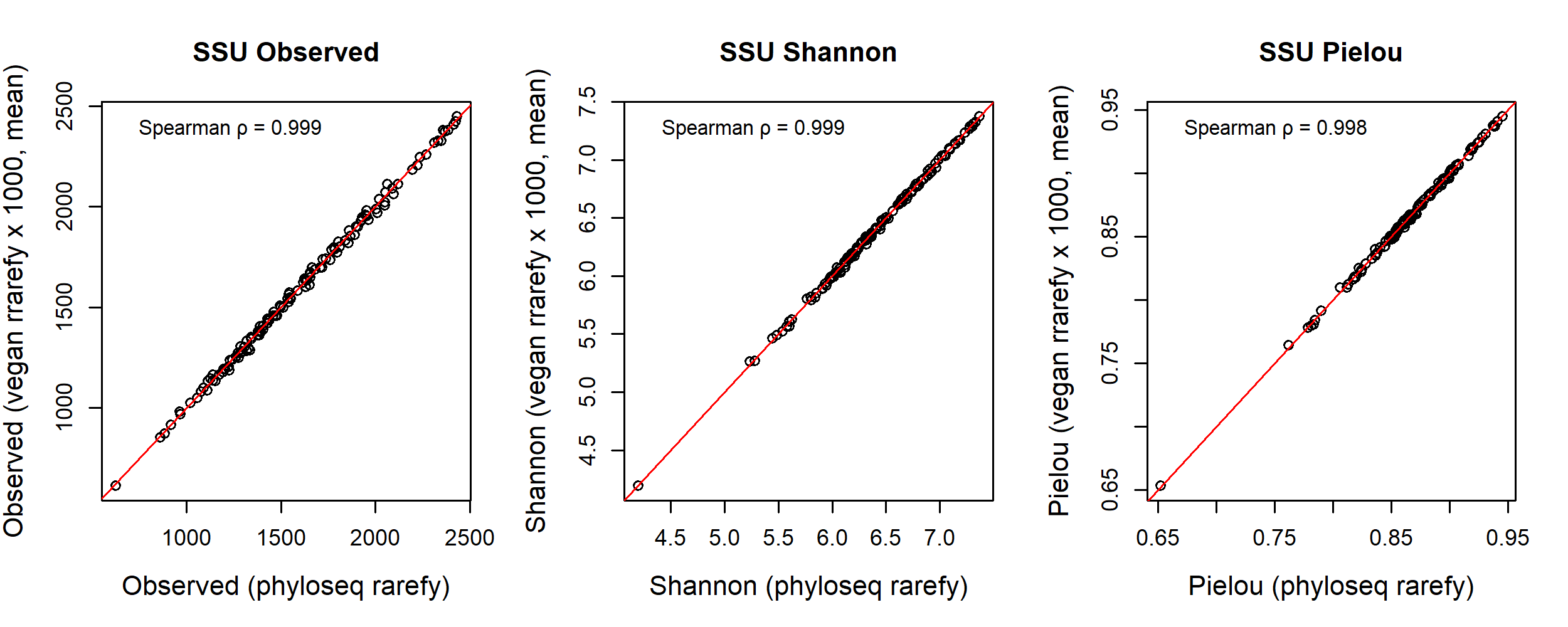


**Figure S2** **|** Comparison of alpha-diversity metrics calculated using two rarefaction approaches. Scatterplots show Observed richness, Shannon diversity and Pielou’s evenness calculated using single rarefaction (*phyloseq::rarefy_even_depth*; x-axis) versus the mean of 1,000 independent rarefactions (*vegan::rrarefy*; y-axis). The strong agreement between methods (Spearman ρ > 0.99) indicates that alpha-diversity estimates are robust to stochastic variation introduced by subsampling.


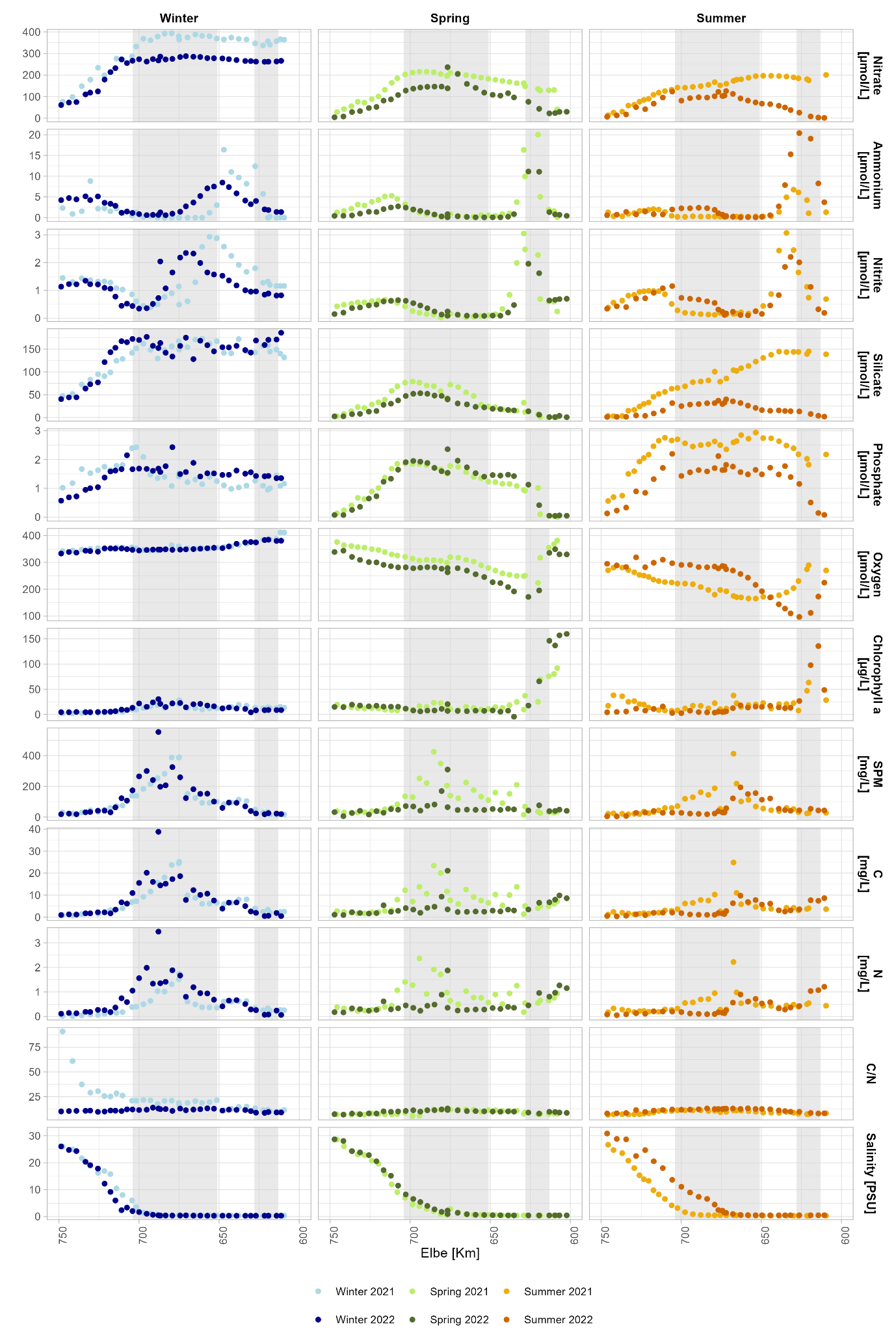


**Figure S3** **|** Longitudinal profiles of the main environmental parameters along the Elbe Estuary (Elbe-km 750–600) during 2021 and 2022. Parameters include nitrate (µmol L-1), ammonium (µmol L-1), nitrite (µmol L-1), phosphate (µmol L-1), silicate (µmol L-1), oxygen (µmol L-1), chlorophyll a (µg L-1), suspended particulate material, particulars C (mg L-1), particulars N (mg L-1), C/N ratio and salinity (PSU). Data are grouped by season (winter, spring, and summer) and year. The grey highlighted areas are the Hamburg Port (613 - 628km) and the MTZ (651 - 704km).


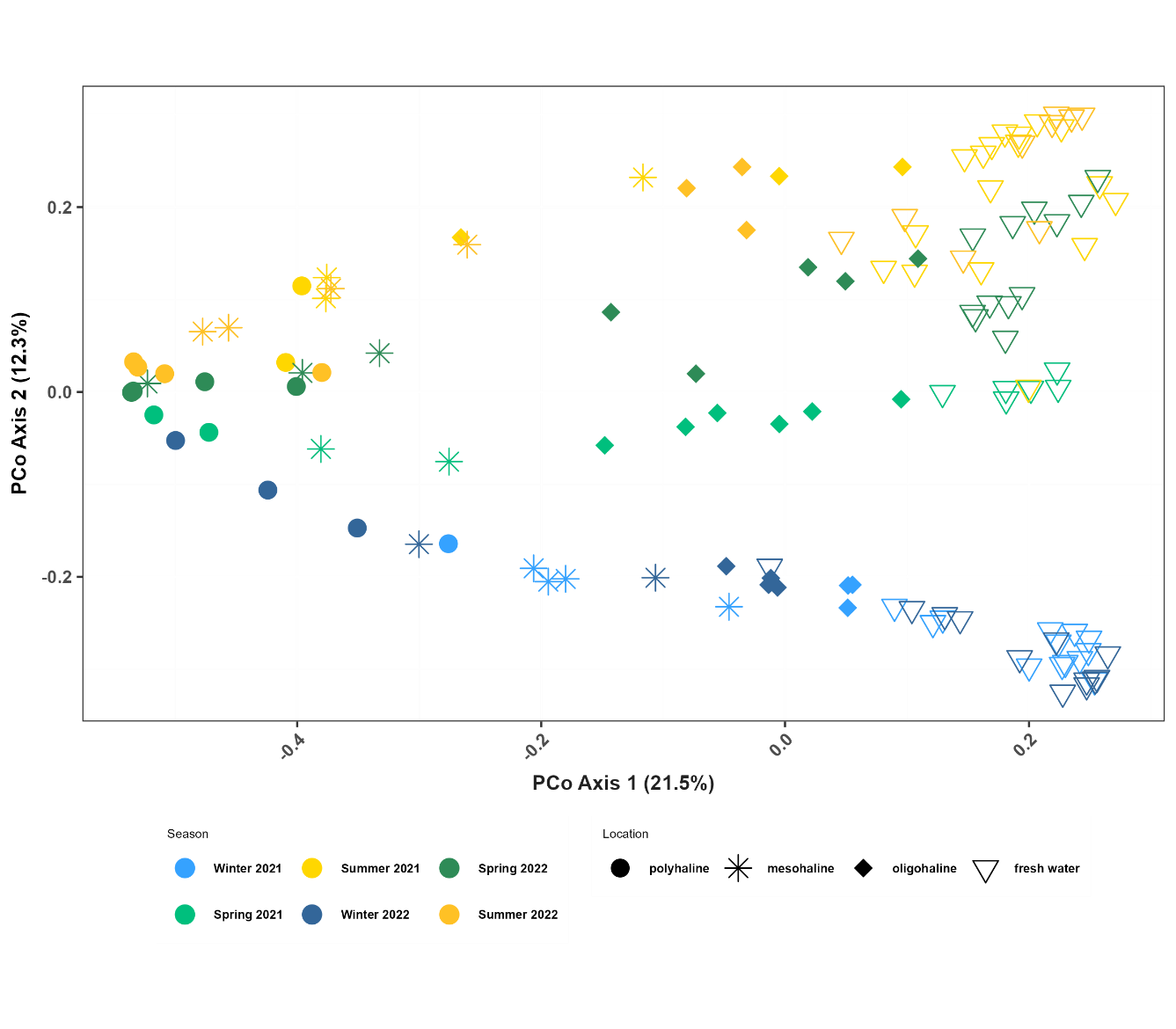


**Figure S4** **|** Principal coordinates analysis (PCoA) was performed based on the Bray–Curtis distances to visualize the composition of bacterial communities at the ASV level.

**Supplementary Material 2 - Table**

**Table S1 |** Pairwise comparisons of Alpha Diversity Indices matrix across environmental categories

The table presents pairwise comparisons of alpha diversity indices across four environmental categories. For each diversity metric (Observed, Chao1, Shannon, Pielou), results from Kruskal-Wallis tests are provided, including test statistics and degrees of freedom, followed by post hoc pairwise comparisons with corresponding Z-scores and adjusted p-values.

**Table S2** | Results of the permutational multivariate analysis of variance (PERMANOVA) including significant pairwise comparisons between levels of salinity, season, region, and year. The Bray-Curtis dissimilarity was used to calculate distance matrices, and multiple comparisons were corrected using the Benjamini–Hochberg method (FDR).

| **Explanatory variable** | **df** | **Pseudo-F** | ***p*-value** | **Multiple comparison** | ***p*-value** |
| --- | --- | --- | --- | --- | --- |
| Salinity | 3 | 14.20403 | **0.001** | Freshwater vs. polyhaline | 0.001 |
|  |  |  |  | Freshwater vs. oligohaline | 0.001 |
|  |  |  |  | Freshwater vs. mesohaline | 0.001 |
|  |  |  |  | Polyhaline vs. oligohaline | 0.001 |
|  |  |  |  | Polyhaline vs. mesohaline | 0.001 |
|  |  |  |  | Oligohaline vs. mesohaline | 0.001 |
| Season | 5 | 7.28241 | **0.001** | Spring 2022 vs. Winter 2021 | 0.001 |
|  |  |  |  | Spring 2022 vs. Spring 2021 | 0.003 |
|  |  |  |  | Spring 2022 vs. Summer 2021 | 0.001 |
|  |  |  |  | Spring 2022 vs. Winter 2022 | 0.001 |
|  |  |  |  | Spring 2022 vs. Summer 2022 | 0.046 |
|  |  |  |  | Winter 2021 vs. Spring 2021 | 0.001 |
|  |  |  |  | Winter 2021 vs. Summer 2021 | 0.001 |
|  |  |  |  | Winter 2021 vs. Winter 2022 | 0.006 |
|  |  |  |  | Winter 2021 vs. Summer 2022 | 0.001 |
|  |  |  |  | Spring 2021 vs. Summer 2021 | 0.001 |
|  |  |  |  | Spring 2021 vs. Winter 2022 | 0.001 |
|  |  |  |  | Spring 2021 vs. Summer 2022 | 0.001 |
|  |  |  |  | Summer 2021 vs. Winter 2022 | 0.001 |
|  |  |  |  | Summer 2021 vs. Summer 2022 | 0.001 |
|  |  |  |  | Winter.2022_vs. Summer.2022 | 0.001 |
| Region | 6 | 8.83329 | **0.001** | A vs. C | 0.001 |
|  |  |  |  | A vs. G | 0.001 |
|  |  |  |  | A vs. F | 0.001 |
|  |  |  |  | A vs. E | 0.001 |
|  |  |  |  | A vs. D | 0.001 |
|  |  |  |  | A vs. B | 0.002 |
|  |  |  |  | C vs. G | 0.001 |
|  |  |  |  | C vs. F | 0.001 |
|  |  |  |  | C vs. E | 0.001 |
|  |  |  |  | C vs. D | 0.005 |
|  |  |  |  | C vs. B | 0.008 |
|  |  |  |  | G vs. F | 0.003 |
|  |  |  |  | G vs. E | 0.001 |
|  |  |  |  | G vs. D | 0.001 |
|  |  |  |  | G vs. B | 0.001 |
|  |  |  |  | F vs. E | 0.002 |
|  |  |  |  | F vs. D | 0.001 |
|  |  |  |  | F vs. B | 0.001 |
|  |  |  |  | E vs. D | 0.016 |
|  |  |  |  | E vs. B | 0.001 |
|  |  |  |  | D vs. B | 0.001 |
| Year | 1 | 4.33748 | **0.001*** |  |  |
| Abbreviations: df – degrees of freedom. Pseudo-f – Pseudo-F statistics.  Note: Significant differences are displayed in bold. | | | | | |

***** The result for *year* should be interpreted with caution, as the assumption of homogeneity of group dispersions was violated (PERMDISP p = 0.048), indicating significant differences in within-group variance. However, results from ANOSIM supported the observed differences (R = 0.06, p = 0.002).

**Table S3** | Distance-based redundancy analysis (dbRDA) shows the relationship between physical–chemical and bacterial community. Adjusted R² (%) indicates variance explained by the model, pseudo-F and p-values (PERMANOVA, n = 999) test the significance of explanatory variables.

| **Ordination axis** | | | |
| --- | --- | --- | --- |
|  | **R^2^_adj._ (%)** | **Pseudo-F** | ***p*-value** |
| Selected Model | 47.33 | 46.069 | 0.001 |
| RDA Axis1 | 17.99 | 27.633 | 0.001 |
| RDA Axis2 | 10.79 | 18.520 | 0.001 |
| **Explanatory variables** | | | |
|  | **Parameter** | **Pseudo-F** | ***p*-value** |
| Variables retained in the model | Discharge | 19.747 | 0.001 |
|  | Salinity | 41.742 | 0.001 |
|  | Temperature | 18.451 | 0.001 |
|  | SiO_4_ | 7.757 | 0.001 |
|  | PO_4_ | 17.372 | 0.019 |
|  | NO_2_ | 4.734 | 0.001 |
|  | Chl *a* | 7.529 | 0.004 |
|  | CN | 3.850 | 0.001 |
